# Modeling and Classification of Kinetic Patterns of Dynamic Metabolic Biomarkers in Physical Activity

**DOI:** 10.1371/journal.pcbi.1004454

**Published:** 2015-08-28

**Authors:** Marc Breit, Michael Netzer, Klaus M. Weinberger, Christian Baumgartner

**Affiliations:** 1 Research Group for Clinical Bioinformatics, Institute of Electrical and Biomedical Engineering (IEBE), University for Health Sciences, Medical Informatics and Technology (UMIT), Hall in Tirol, Austria; 2 sAnalytiCo Ltd, Belfast, United Kingdom; 3 Institute of Health Care Engineering with European Notified Body of Medical Devices, Graz University of Technology, Graz, Austria; Hellas, GREECE

## Abstract

The objectives of this work were the classification of dynamic metabolic biomarker candidates and the modeling and characterization of kinetic regulatory mechanisms in human metabolism with response to external perturbations by physical activity. Longitudinal metabolic concentration data of 47 individuals from 4 different groups were examined, obtained from a cycle ergometry cohort study. In total, 110 metabolites (within the classes of acylcarnitines, amino acids, and sugars) were measured through a targeted metabolomics approach, combining tandem mass spectrometry (MS/MS) with the concept of stable isotope dilution (SID) for metabolite quantitation. Biomarker candidates were selected by combined analysis of maximum fold changes (MFCs) in concentrations and *P*-values resulting from statistical hypothesis testing. Characteristic kinetic signatures were identified through a mathematical modeling approach utilizing polynomial fitting. Modeled kinetic signatures were analyzed for groups with similar behavior by applying hierarchical cluster analysis. Kinetic shape templates were characterized, defining different forms of basic kinetic response patterns, such as sustained, early, late, and other forms, that can be used for metabolite classification. Acetylcarnitine (C2), showing a late response pattern and having the highest values in MFC and statistical significance, was classified as late marker and ranked as strong predictor (MFC = 1.97, *P* < 0.001). In the class of amino acids, highest values were shown for alanine (MFC = 1.42, *P* < 0.001), classified as late marker and strong predictor. Glucose yields a delayed response pattern, similar to a hockey stick function, being classified as delayed marker and ranked as moderate predictor (MFC = 1.32, *P* < 0.001). These findings coincide with existing knowledge on central metabolic pathways affected in exercise physiology, such as β-oxidation of fatty acids, glycolysis, and glycogenolysis. The presented modeling approach demonstrates high potential for dynamic biomarker identification and the investigation of kinetic mechanisms in disease or pharmacodynamics studies using MS data from longitudinal cohort studies.

## Author Summary

Human metabolism is controlled through basic kinetic regulatory mechanisms, where the overall system aims to maintain a state of homeostasis. In response to external perturbations, such as environmental influences, nutrition or physical exercise, circulating metabolites show specific kinetic response patterns, which can be computationally modeled. In this work, we searched for dynamic metabolic biomarker candidates and analyzed specific kinetic mechanisms from longitudinal metabolic concentration data, obtained through a cycle ergometry stress test. In total, 110 metabolites measured from blood samples of 47 individuals were analyzed using tandem mass spectrometry (MS/MS). Dynamic biomarker candidates could be selected based on the amplitudes of changes in metabolite concentrations and the significance of statistical hypothesis testing. We were able to characterize specific kinetic patterns for groups of similarly behaving metabolites. Kinetic shape templates were identified, defining basic kinetic response patterns to physical exercise, such as sustained, early, late and other shape forms. The presented approach contributes to a better understanding of (patho)physiological biochemical mechanisms in human health, disease or during drug therapy, by offering tools for classifying dynamic biomarker candidates and for modeling and characterizing kinetic regulatory mechanisms from longitudinal experimental data.

## Introduction

### Metabolite kinetics—biochemical aspects

Basic principles in reaction kinetics of biomolecules were described by the work of Guldberg & Waage [1–3] more than 150 years ago and recently resumed by Voit *et al*., 2015 [4] in their perspective article "150 years of mass action". The underlying concept is the law of mass action, describing the quantitative aspects of a chemical reaction under ideal conditions. If a substance C is formed by the reaction of substance A and substance B, the production of C can be described by the following equation

ProductC=k*A*B (1)

where *A*, *B*, and *C* are concentrations changing over time, and *k* is a rate constant describing the speed of the reaction. Probably the most widely known and used modification of the original model in biochemistry is the Michaelis-Menten rate law (MMRL) introduced by Michaelis & Menten in 1913 [5]

$$v=\;\frac{V_{max}\;S}{K_{m}+S}$$ (2)

where *v* is the reaction rate, *V*_*max*_ the maximum reaction rate, *S* the concentration of the substrate, and *K*_*m*_ the Michaelis constant (the substrate concentration at half of the maximum reaction rate).

The Michaelis-Menten model describes the reaction kinetics of an enzyme-catalyzed single-substrate reaction, in which the conversion of a substrate *S* into a product *P* takes place via the formation of an intermediate complex *ES*, where *k*_*1*_, *k*_*2*_ and *k*_*3*_ denote reaction rates [4]

$$E+S\begin{array}{c} \overset{\;k_{2}\;}{\leftarrow }\\ \underset{\;k_{1}\;}{\to } \end{array}ES\overset{\;k_{3}\;}{\to }E+P$$ (3)

Guldberg and Waage also examined the fact that biochemical systems tend to remain in homeostasis, which is described by the equilibrium constant [6]

$$K_{eq}=\frac{{\left[\text{C}\right]}^{\text{c}}{\left[\text{D}\right]}^{\text{d}}}{{\left[\text{A}\right]}^{\text{a}}{\left[\text{B}\right]}^{\text{b}}}$$ (4)

*K*_*eq*_ is the equilibrium constant in the general reaction *aA + bB*↔*cC + dD*, where *a*, *b*, *c*, *d* are the number of molecules of *A*, *B*, *C*, *D* participating, and *[A]*, *[B]*, *[C]*, *[D]* are the molar reaction concentrations of the reaction components at equilibrium [7].

When analyzing regulatory mechanisms of metabolite kinetics, a key question addresses the effect of external perturbations disturbing homeostasis, e.g., caused by environmental influences, nutrition, drug interventions (pharmacodynamics) or physical activity (studied in this work through clinical exercise testing). These effects can be measured and examined by longitudinal cohort studies, which investigate dynamic changes in metabolite concentrations over time.

In chronic toxicity testing, which occupies a central position in the analysis of dynamic time-course metabolic data, studies are performed to explore the influences of toxic substances on human or animal metabolism. Mechanisms of metabolite kinetics are analyzed, e.g., by investigating the effect of pesticide exposure on children [8,9], by *in-vitro* examination of drug induced effects in neurotoxicity using brain cell cultures [10], or by analysis of toxic effects of polymers or nanoparticles to the water flea *daphnia magna* [11,12]. In biotechnological process monitoring, metabolic interactions are analyzed, e.g., in studying the sensitivity of the biocatalyst *Clostridium thermocellum* to ethanol stress [13], in exploring the forced ageing process of Port wine [14] or by the examination and optimization of cell culture media, as e.g., of Chinese hamster ovary (CHO) cells [15–17]. In pharmacodynamics, time-course data are collected, e.g., to study the effect of continuous exposure of breast cancer cells to an anti-cancer chemotherapy drug on the metabolic level [18] or to explore the metabolism of albumin in patients with systemic inflammatory response syndrome after continuous venovenous hemofiltration [19]. Research questions on kinetic mechanisms in physical exercise cover fundamental work, e.g., on studying the influence of improved metabolic health on patterns in plasma metabolites [20] or analyzing the effects of aerobic exercise on oral glucose tolerance [21].

In this work, in response to an incrementally increased physical load by cycle ergometry and depending on the underlying metabolic regulatory mechanisms, metabolites are expected to show specific kinetic signatures and shape patterns. Expected kinetic response patterns include: a sustained response (mainly constant concentration over time, overlaid with biological or instrumental noise), an early response (main decrease/increase of concentrations shortly after start of activity), a halving interval response (major change in concentration at half time of physical activity, e.g., a sigmoid behavior with a plateau), a late response (strongest decrease/increase of concentration towards the end of physical activity), and a delayed response pattern (first mainly sustained metabolite concentration, then showing a strong reaction after the end of activity during the recovery phase, respectively).

### Modeling of kinetic mechanisms—computational aspects

Regarding computational and mathematical aspects of characterizing kinetic regulatory mechanisms, different approaches of fundamental models for the analysis of metabolic processes have been described in the literature: qualitative models for topological network analysis, models of flux balance analysis using stoichiometric network construction and detailed kinetic models representing metabolic processes using ordinary differential equations (ODEs) [22,23]. Furthermore, different intermediate approaches do exist, e.g., the approach of structural kinetic modeling (SKM), approximating local biochemical mechanisms within a metabolic network by a parametric linear representation [23]. An overview on different "approximative kinetic formats used in metabolic network modeling" is given by Heijnen, 2005 [24]. An example for the dynamic simulation of kinetic mechanisms in metabolism—by simulating the mitochondrial fatty acid β-oxidation—is presented by Modre *et al*., 2009 [25]. Further examples for theoretical network models as well as dynamic kinetic simulations can be found in the context of the e-cell project [26], e.g., models for drosophila [27] or the metabolic simulation of red blood cell storage [28].

With respect to the analysis of dynamic metabolic data, Smilde *et al*., 2010 [29] distinguish between six groups of methods: methods based on fundamental models, predefined basic functions, dimension reduction, multivariate time series models, analysis-of-variance (ANOVA) type models, and methods based on imposing smoothness. Analyses of periodic or oscillating data can be performed using methods such as Fourier analysis, wavelet transformation or principal component analysis (PCA) with wavelets [29,30]. Hidden Markov models were presented as a way for using basic functions, allowing flexibility and adaptation in modeling [29,31]. In particular, in gene-expression analysis orthogonal polynomials were introduced for qualitative and quantitative modeling [32,33].

Alternative methods for the analysis of longitudinal metabolic data, typically used in nuclear magnetic resonance (NMR) spectroscopy, comprise weighted principal component analysis (WPCA) [34] or analysis of variance (ANOVA) simultaneous component analysis (ASCA) [35]. A statistical framework for metabolic biomarker discovery in NMR data was presented by Berk *et al*., 2011 [36], introducing a smoothing spline mixed effects (SME) model, combined with an associated functional test statistic. Mishina *et al*., 1993 [37] suggested analyzing the kinetics of biomolecules by fitting differential equations for the application in pharmacodynamics. A method for investigating between-metabolite relationships by simultaneous component analysis with individual differences constraints (SCA-IND) was presented by Jansen *et al*., 2012 [38]. A new method for combined analysis of proteomics and metabolomics data using integrative pathway analysis was introduced by Stanberry *et al*., 2013 [39]. As an example for a web-based, freely accessible online service, Metaboanalyst [40] offers the profiling of longitudinal time-course data on the basis of a multivariate empirical Bayes approach.

### Metabolic biomarker discovery

Metabolic biomarkers play an essential role in clinical diagnostics because of their ability to provide specific insights by being functional endpoints of human molecular interactions [41]. The general process for the discovery, verification, and validation of metabolic biomarker candidates was described by Baumgartner & Graber, 2008 [42]. This process ranges from experimental study design, over clinical study execution, execution of bioanalytical methods and acquisition of data, consolidation and integration of data, application of bioinformatics algorithms and data mining methods for the identification of biomarker candidates, up to an independent validation of putative biomarkers by clinical trials. In their review article, Baumgartner *et al*., 2011 [43] give a comprehensive survey of computational data analysis strategies for the discovery of biomarker candidates from metabolic data.

A milestone in clinical application of metabolic biomarkers was set by establishing routine newborn screening programs for inherited metabolic disorders [44]. The search for novel metabolic biomarkers in disease covers a wide range of clinical application areas, e.g., the identification of metabolic markers in prostate cancer by a rule-based feature selection algorithm [45], the search of early markers as well as late markers in planned and spontaneous myocardial infarction [46,47], the investigation of metabolic mechanisms in diabetes [48–50] or the discovery of putative biomarker candidates in chronic kidney disease [51–53].

### Research objectives

In this article, we present a computational methodology, aimed at the modeling and characterization of kinetic regulatory mechanisms and the discovery of dynamic metabolic biomarker candidates in physical activity. Dynamic time-course metabolic concentration data are generated from a longitudinal biomarker cohort study by standardized cycle ergometry experiments. In total, 110 metabolites (including metabolite classes of acylcarnitines, amino acids and sugars) are quantitated by a targeted metabolomics approach utilizing mass spectrometry. After a thorough examination of the measured concentration data in terms of data quality assurance and reliability, we selected a set of 30 metabolites relevant in exercise physiology and considered them for data analysis and modeling in this work.

Metabolite concentrations of 47 individuals, showing different lengths in their concentration time curves (depending on the individual’s maximum physical load), are made comparable by means of data preprocessing. Biomarker candidates are selected depending on maximum fold changes (MFCs) (the amplitude of changes in concentrations) and the corresponding *P*-values resulting from statistical hypothesis testing. Kinetic signatures of metabolites are quantified by a mathematical modeling approach using polynomial fitting, specifying the dynamic response patterns of analyzed metabolites during physical activity. A similarity measure for characterized metabolite kinetic signatures is obtained through specification of groups of metabolites by hierarchical cluster analysis. Kinetic shape templates are identified, specifying common kinetic response patterns and enabling the classification of dynamic metabolic biomarker candidates according to their kinetic patterns. Findings are verified and interpreted through biochemical and metabolic pathway analyses associated with physical activity.

## Results

### Selection of dynamic biomarker candidates

**Fig 1:**
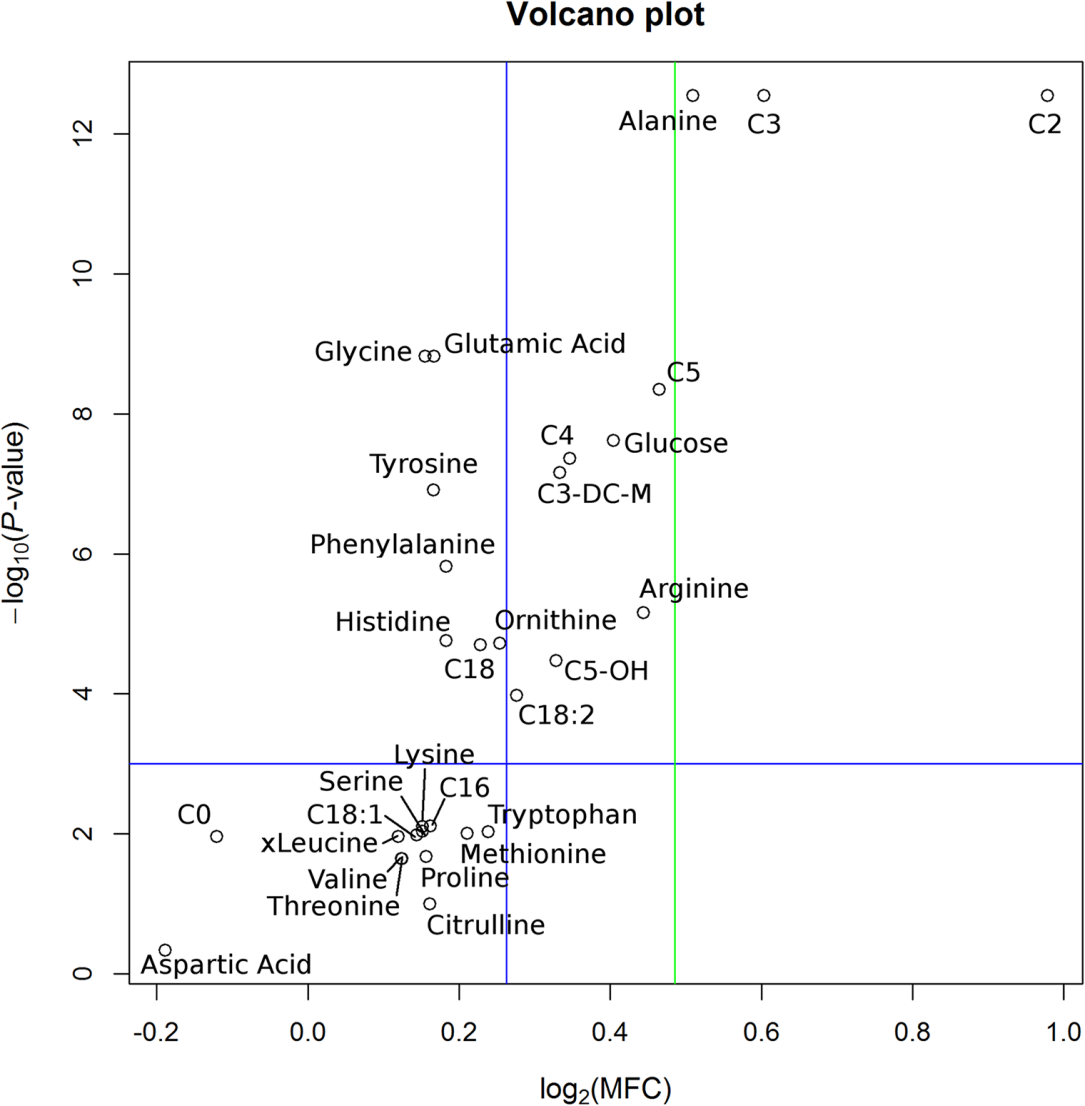
Volcano plot. The volcano plot displays the log_2_(MFC) between minimum and maximum concentrations values versus the-log_10_(*P*-value) calculated from statistical hypothesis testing. The horizontal blue line indicates the selected significance level of 0.001. The vertical blue line indicates the threshold for classification as moderate predictor (MFC > 1.20). The vertical green line denotes the classification threshold for a strong predictor (MFC > 1.40). Acetylcarnitine (C2), propionylcarnitine (C3) and alanine could be selected as strong biomarker candidates. Valerylcarnitine (C5), arginine, glucose, butyrylcarnitine (C4), methylmalonylcarnitine (C3-DC-M), and hydroxyvalerylcarnitine (C5-OH) were identified as moderate biomarker candidates.

Putative dynamic biomarker candidates are selected from the pool of analyzed metabolites by combined analysis of MFCs in concentrations and corresponding *P*-values from statistical hypothesis testing (see section Maximum fold changes and statistical hypothesis testing). Results for this data analysis step are visualized as a volcano plot (Fig 1). The plot demonstrates log_2_ values of MFCs compared to-log_10_ values of *P*-values. A significance level of 0.001 was chosen for the selection of statistical hypothesis testing results (horizontal blue line). Moderate biomarker candidates are classified with a MFC greater than 1.20 (vertical blue line). Strong biomarker candidates are classified with a MFC greater than 1.40 (vertical green line). Detailed data of all analyzed metabolites, including MFCs, log_2_(MFCs), *P*-values, and-log_10_(*P*-values), are summarized in Table 1.

**Table 1:** Selection of dynamic biomarker candidates.

Metabolite	MFC	log_2_(MFC)	*P*-value	-log_10_(*P*-value)
Acetylcarnitine (C2)	1.97	0.98	2.84E-13	12.55
Propionylcarnitine (C3)	1.52	0.6	2.84E-13	12.55
Alanine	1.42	0.51	2.84E-13	12.55
Valerylcarnitine (C5)	1.38	0.46	4.49E-09	8.35
Arginine	1.36	0.44	6.85E-06	5.16
Glucose	1.32	0.4	2.38E-08	7.62
Butyrylcarnitine (C4)	1.27	0.35	4.31E-08	7.37
Methylmalonylcarnitine (C3-DC-M)	1.26	0.33	6.93E-08	7.16
Hydroxyvalerylcarnitine (C5-OH)	1.26	0.33	3.35E-05	4.48
Octadecadienylcarnitine (C18:2)	1.21	0.28	1.05E-04	3.98
Ornithine	1.19	0.25	1.87E-05	4.73
Tryptophan	1.18	0.24	9.28E-03	2.03
Octadecanoylcarnitine (C18)	1.17	0.23	1.97E-05	4.71
Methionine	1.16	0.21	9.81E-03	2.01
Histidine	1.14	0.18	1.72E-05	4.76
Phenylalanine	1.14	0.18	1.51E-06	5.82
Hexadecanoylcarnitine (C16)	1.12	0.16	7.68E-03	2.11
Citrulline	1.12	0.16	9.91E-02	1.00
Glutamic_Acid	1.12	0.17	1.48E-09	8.83
Tyrosine	1.12	0.17	1.21E-07	6.92
Glycine	1.11	0.15	1.48E-09	8.83
Lysine	1.11	0.15	7.77E-03	2.11
Proline	1.11	0.16	2.09E-02	1.68
Serine	1.11	0.15	9.10E-03	2.04
Octadecenoylcarnitine (C18:1)	1.10	0.14	1.04E-02	1.98
Threonine	1.09	0.12	2.25E-02	1.65
Valine	1.09	0.12	2.25E-02	1.65
xLeucine (Leucine + Isoleucine)	1.09	0.12	1.08E-02	1.97
Carnitine (C0)	0.92	-0.12	1.08E-02	1.97
Aspartic_Acid	0.88	-0.19	4.55E-01	0.34

For selection of dynamic biomarker candidates, MFC, log_2_(MFC), *P*-value, and-log_10_(*P*-value) are summarized for each metabolite in the table. The majority of metabolites show a positive value for log_2_(MFC), where only two metabolites, namely carnitine (C0) and aspartic acid, show a negative value. Identical *P*-values, shown for some metabolites, result from the applied statistical test (Wilcoxon Signed Rank test for paired samples), which uses ranks for hypothesis testing. *P*-values are adjusted according to the false discovery rate (FDR) correction for multiple comparisons (see section Maximum fold changes and statistical hypothesis testing).

For the analyzed classes of metabolites, putative biomarker candidates could be selected and ranked according to MFCs and *P*-values. As strong biomarker candidates, acetylcarnitine (C2, MFC = 1.97, *P* <0.001), showing the highest values in the entire set of analyzed metabolites, propionylcarnitine (C3, MFC = 1.52, *P* < 0.001) and alanine (MFC = 1.42, *P* < 0.001) were identified. Valerylcarnitine (C5, MFC = 1.38, *P* < 0.001), arginine (MFC = 1.36, *P* < 0.001), glucose (MFC = 1.32, *P* < 0.001), butyrylcarnitine (C4, MFC = 1.27, *P* < 0.001), methylmalonylcarnitine (C3-DC-M, MFC = 1.26, *P* < 0.001), hydroxyvalerylcarnitine (C5_OH, MFC = 1.26, *P* < 0.001), and octadecadienylcarnitine (C18:2, MFC = 1.21, *P* < 0.001) were ranked as moderate biomarker candidates.

### Characterization of metabolite kinetic signatures

**Fig 2:**
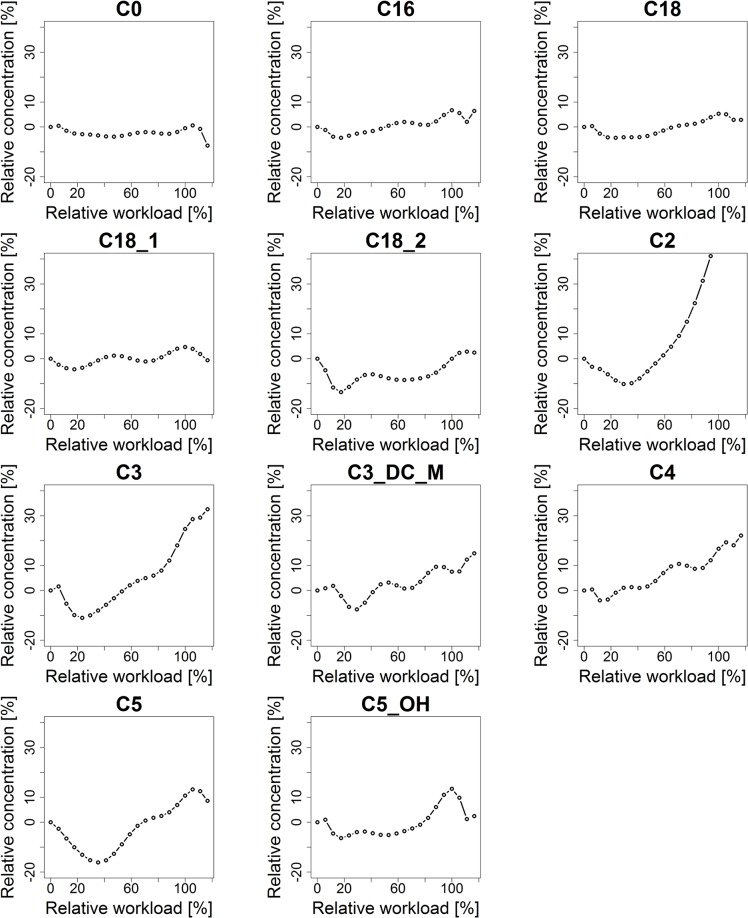
Kinetic signatures of acylcarnitines. Kinetic signatures of the 11 selected acylcarnitines are depicted. Dynamic curves were characterized by polynomial fitting of 9^th^ degree to the median concentration values of the analyzed metabolites. For visualization, relative changes (in %) of metabolite concentrations in reference to their initial concentration at rest are displayed. An early response pattern is shown for valerylcarnitine (C5) with a decrease in relative concentration of approx. 16%. Late response profiles include acetylcarnitine (C2), propionylcarnitine (C3) and butyrylcarnitine (C4).

**Fig 3:**
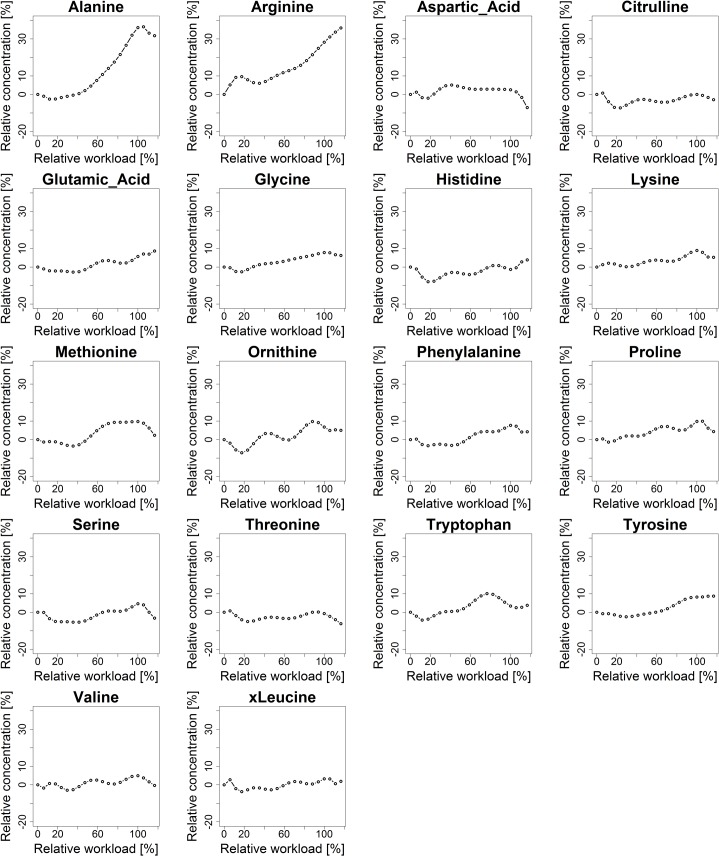
Kinetic signatures of amino acids. Kinetic signatures of the 18 selected amino acids. Methionine yields a halving interval response pattern with a plateau (sigmoid characteristics). Alanine and arginine show a late response pattern.

**Fig 4:**
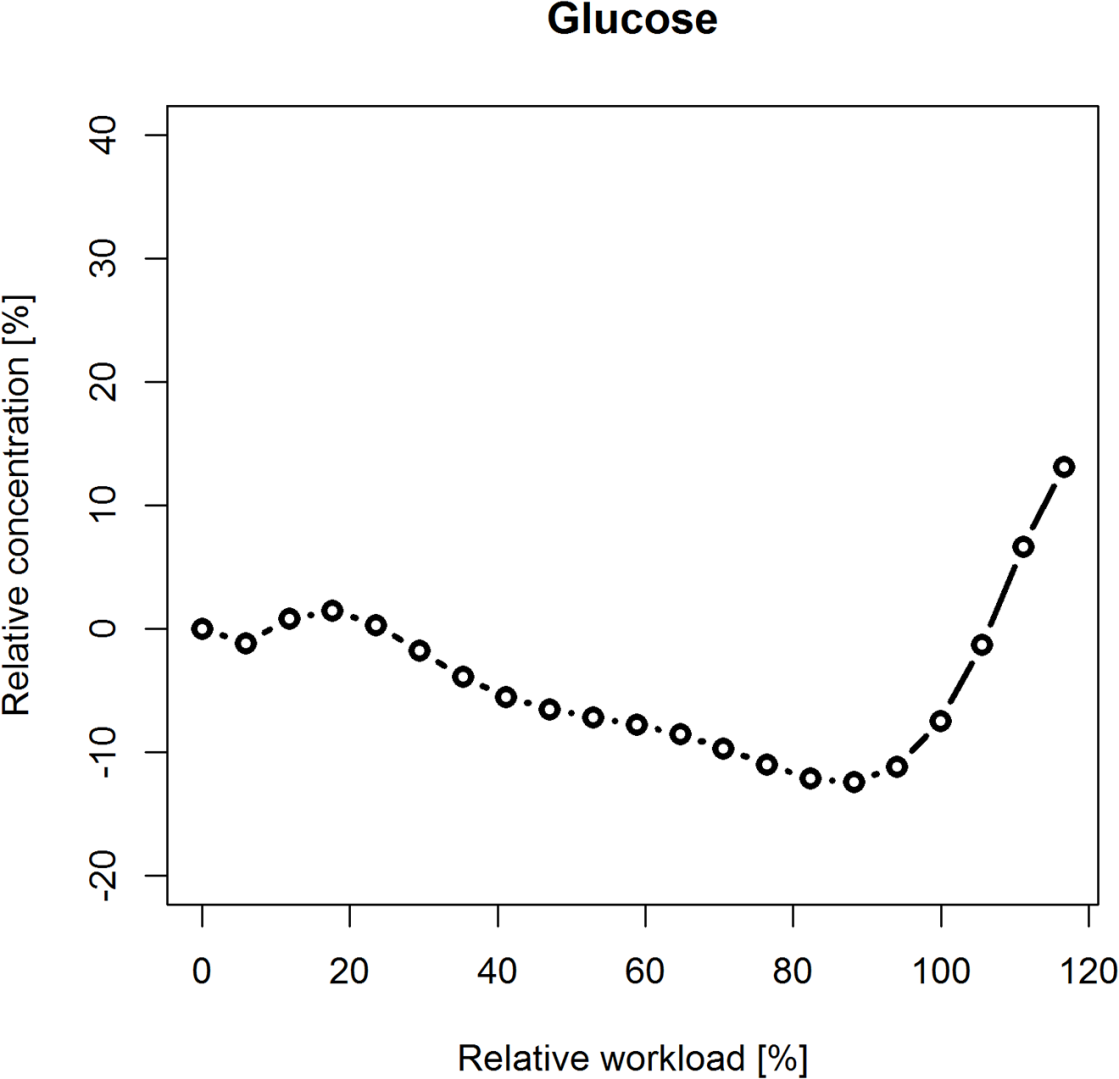
Kinetic signature of glucose. A delayed response pattern is apparent in glucose, decreasing in relative concentration (-12%) towards the end of exercise with a steep increase (up to 13%) after the end of exercise during the recovery phase.

Kinetic signatures of analyzed metabolites are expected to show specific characteristic regulatory patterns, in response to the incremental increase of physical activity using a cycle ergometry stress test. Kinetic patterns of the selected 30 metabolites, characterized by a polynomial fitting approach (see section Mathematical modeling), are visualized in Fig 2 (acylcarnitines), Fig 3 (amino acids) and Fig 4 (glucose). For standardized visualization of profiles, the vertical axis is normalized to a range of-20% to 40% of relative concentration. Note that acetylcarnitine (C2) exceeds this specified range, showing a maximum increase in relative concentration of 67%.

Different kinetic response patterns were observed. The majority of metabolites show a sustained response, e.g., threonine, with basically constant behavior in concentration over time, however overlaid with biological or instrumental noise. An early response pattern is shown with valerylcarnitine (C5) with an early decrease in relative concentration (of approx. -16%) after starting exercise followed by an increase in relative concentration (to a maximum of 13%). Methionine could be identified as a metabolite showing a halving interval response pattern with characteristics similar to a sigmoid function, showing first a sustained reaction, then an increase in relative concentration at half time of physical activity (by approx. 13%) and followed by a plateau (at approx. 9% of relative concentration) towards the end of physical exercise. Metabolites showing a late response pattern are e.g., acetylcarnitine (C2) with a slight decrease (-10%) and then a strong continuous increase in relative concentration (up to 67%) or alanine with a continuous increase (of approx. 32%) up to the end of exercise. Glucose shows a delayed response pattern (similar to a L-curve / hockey stick function, see section Mathematical modeling) with a minor increase in relative concentration (approx. 2%) at the beginning of exercise, followed by a continuous decrease (down to-12%) and a major steep increase in relative concentration (up to 13%) after the end of exercise during the recovery phase.

### Identification of metabolite groups with similar patterns

Groups of metabolites, showing similar kinetic patterns with response to physical exercise, were identified by hierarchical cluster analysis (see section Hierarchical cluster analysis), resulting in a set of seven distinct clusters. Metabolites and their corresponding cluster affiliations are summarized in Table 2.

**Table 2:** Metabolite groups with similar patterns.

Metabolite	Cluster identifier
Alanine	1
Arginine	1
Aspartic Acid	2
Carnitine (C0)	2
Octadecanoylcarnitine (C18)	2
Octadecenoylcarnitine (C18:1)	2
Hydroxyvalerylcarnitine (C5-OH)	2
Citrulline	2
Histidine	2
Serine	2
Threonine	2
Valine	2
xLeucine (leucine + isoleucine)	2
Hexadecanoylcarnitine (C16)	3
Methylmalonylcarnitine (C3-DC-M)	3
Glutamic Acid	3
Glycine	3
Lysine	3
Methionine	3
Ornithine	3
Phenylalanine	3
Proline	3
Tryptophan	3
Tyrosine	3
Octadecadienylcarnitine (C18:2)	4
Glucose	4
Acetylcarnitine (C2)	5
Propionylcarnitine (C3)	6
Butyrylcarnitine (C4)	6
Valerylcarnitine (C5)	7

Metabolites and their cluster affiliations which were identified by hierarchical cluster analysis. Cutting the hierarchical tree at a threshold of 35 results in 7 different clusters.

Cluster 1 consists of the two amino acids alanine and arginine. Cluster 2 and cluster 3 comprise a multitude of metabolites of similar metabolite kinetics, which show roughly sustained response patterns. In cluster 4, the metabolites octadecadienylcarnitine (C18:2) and glucose are clustered together. Cluster 5 consists of only acetylcarnitine (C2), the metabolite showing the strongest response. In cluster 6, propionylcarnitine (C3) and butyrylcarnitine (C4) are grouped together. Cluster 7 represents valerylcarnitine (C5), a biomarker candidate showing an early response pattern.

### Specification of kinetic shape templates

**Fig 5:**
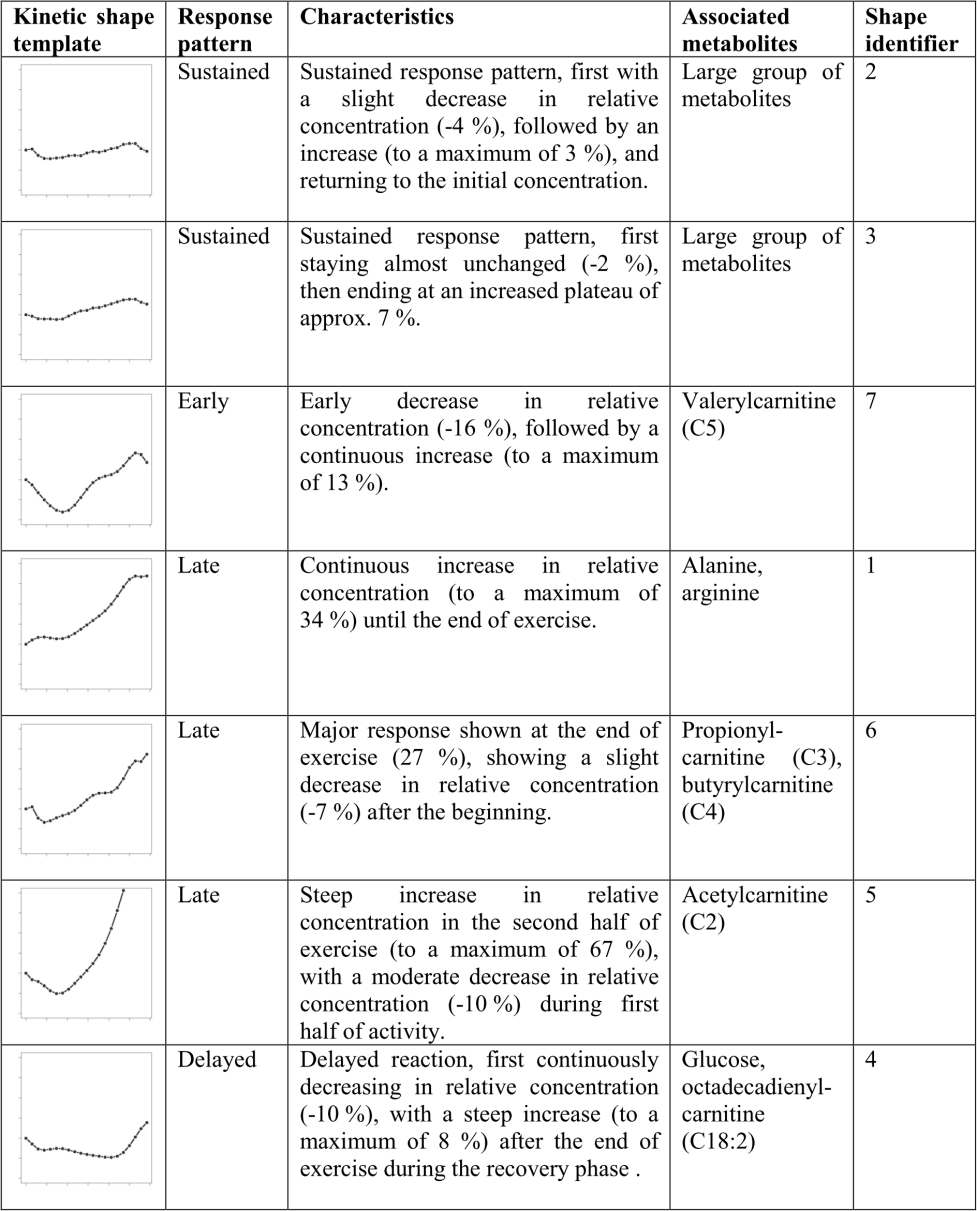
Kinetic shape templates. Kinetic shape templates for the classification of similar dynamic patterns.

Kinetic shape templates, serving for the classification of similar metabolite dynamics, could be specified based on the median concentration curves of each identified cluster (see section Hierarchical cluster analysis). Identified shapes and their characteristics are summarized in Fig 5, based on relative concentration changes in reference to the initial concentration at rest. Identifiers of kinetic shape templates hereby correspond to identifiers of resulting metabolite clusters from hierarchical cluster analysis.

Templates for sustained response patterns, observed in the majority of metabolites, are specified by shapes 2 and 3. Shape 7 specifies a template for dynamic biomarker candidates, showing an early response pattern (valerylcarnitine (C5)). Shape 1 describes a template showing a late response pattern with a continuous increase in concentration (alanine and arginine). Shapes 5 (acetylcarnitine (C2)) and 6 (propionylcarnitine (C3) and butyrylcarnitine (C4)) define further templates for late response patterns, differing in their dynamics in concentration time courses and maximum concentration changes. Shape 4 demonstrates a template for a delayed response pattern, showing characteristics similar to a L-curve / hockey-stick function (glucose and octadecadienylcarnitine (C18:2)).

### Classification of dynamic biomarker candidates

Dynamic metabolic biomarker candidates are identified and classified through a two-step analysis procedure: first, by analysis of MFCs in concentrations and statistical hypothesis testing, and second, by reviewing and characterizing specified metabolic response patterns and kinetic shape templates.

The majority of metabolites show a sustained response pattern, staying within an interval of relative MFC of less than 20%, being ineligible as putative biomarker candidates. Valerylcarnitine (C5), yielding an early response pattern, was classified as early marker and moderate predictor (MFC = 1.38, *P* < 0.001). Methionine shows a halving interval response pattern with a sigmoid behavior, but having a moderate amplitude in concentration (MFC = 1.16, P > 0.001) and was therefore not selected as a biomarker candidate. A late response pattern with weak early decrease in concentration was observed with propionylcarnitine (C3) (strong predictor, MFC = 1.52, *P* < 0.001), and butyrylcarnitine (C4) (moderate predictor, MFC = 1.27, *P* < 0.001), both classified as late biomarker candidates. Alanine (strong predictor, MFC = 1.42, *P* < 0.001) and arginine (moderate predictor, MFC = 1.36, *P* < 0.001) showed a late response pattern with a continuous increase in concentration from the beginning of exercise and were classified as late markers. Highest concentration changes yielded acetylcarnitine (C2), demonstrating a late response pattern with a very strong increase towards the end of exercise. C2 was ranked as strong predictor (MFC = 1.97, *P* < 0.001) and classified as late marker. Showing basic delayed response patterns, glucose (MFC = 1.32, *P* < 0.001) and octadecadienylcarnitine (C18:2) (MFC = 1.21, *P* < 0.001) were identified as moderate predictors and classified as delayed markers.

### Biochemical interpretation of findings

Thanks to an elaborate body of knowledge in biochemistry, a peculiarity within the process of data analysis in metabolomics lies in the dedicated biochemical interpretation of results [54]. This knowledge is nowadays annotated in public databases, e.g., the Kyoto Encyclopedia of Genes and Genomes (KEGG) [55], and eases the interpretation of findings in the context of annotated biochemical pathways.

In exercise physiology, various biochemical reactions in metabolism play an essential role, predominantly in carbohydrate metabolism (glycolysis and glycogenolysis), in lipid metabolism (β-oxidation of free fatty acids) and amino acid metabolism [6]. During a cycle ergometry stress test, an individual increasingly consumes adenosine triphosphate (ATP); to compensate this energy consumption and maintain homeostatic levels of ATP, its production is up-regulated, first primarily by aerobic processes (respiration), and then anaerobic fermentation. Under the low-impact conditions chosen in this study (low initial output of 50 Watt (W) and slow increase of 25 W every three minutes), the metabolic data demonstrate that the body uses both glycolysis and β-oxidation of fatty acids as readily available energy sources, before protein catabolism contributes in a substantial manner. Of course, the pools of monosaccharides and free fatty acids have to be replenished by glycogenolysis and lipolysis, respectively.

The findings of this work, i.e., identified biomarker candidates of exercise metabolism, and characterized metabolite signatures via specified kinetic shape templates, can be explained through the metabolic regulatory mechanisms in physical activity. Significant changes in concentrations of acetylcarnitine (C2) and closely related short-chain acylcarnitines (C3, C4, and C5) arise from their involvement in the β-oxidation of free fatty acids with acetylcarnitine (the single most significant finding) representing the actual end-point of the β-oxidation of even-numbered fatty acids which constitute the vast majority of dietary fatty acids and of fatty acids in the body's adipose tissue. The strong increase in concentrations of alanine and arginine are representative for an increased production of glucogenic amino acids through high glycolytic activity. This connection is most obvious for alanine, which is the corresponding amino acid of the alpha-keto acid pyruvate and is, thus, a direct mirror of glycolytic or gluconeogenetic flux [48]. The third major finding, the overproduction of glucose after the end of the exercise, is due to the inertia of metabolic regulation. In order to supply the glycolysis with enough fuel, glucose has to be released from its storage by glycogenolysis. At the abrupt end of the exercise, the increased activity of the glycogenolytic machinery cannot be stopped immediately and, therefore, leads to overcompensated glucose levels.

## Discussion

### Summary

In this work we have presented a computational modeling and statistics approach for the identification of dynamic metabolic signatures through characterization of kinetic patterns of circulating metabolites from a physical exercise study. Dynamic time-course metabolic concentration data were obtained through clinical exercise testing using a cycle ergometry stress test.

The data of 47 individuals from four different groups were analyzed: male and female test persons, with either average physical activity or competitive athletic activity. Lactate concentrations were measured for all individuals as a gold standard for profiling physical activity. Metabolite concentrations were quantitated by a targeted metabolomics approach, combining mass spectrometry analytics with the concept of stable isotope dilution. From the initial set of 110 metabolites (including classes of acylcarnitines, amino acids and sugars), we selected a reliable and quality assured set of 30 metabolites for data analysis playing a possible role in exercise physiology.

Based on the generic process for biomarker discovery in metabolomics, a computational approach for the analysis of longitudinal metabolic concentration data was developed. Computational tools were implemented in R [56] for automating the data analysis workflow. The source file (R script file) and the underlying dataset (Microsoft Excel file) are provided as supporting information (S1 File and S2 File). Individual workload curves, differing in the number of measurements due to variability in the individual's physical capacity and exertion, were made comparable by data preprocessing steps including rescaling and linear interpolation of concentration-time curves.

Putative dynamic biomarker candidates for physical activity were selected by combined analysis of MFCs in concentrations and *P*-values of statistical hypothesis testing. Kinetic patterns of analyzed metabolites were characterized based on a mathematical modeling approach utilizing polynomial fitting as the method of choice. Metabolite groups, showing similar kinetic response patterns, were obtained by applying hierarchical cluster analysis to the set of characterized metabolite kinetic patterns. Kinetic shape templates could be specified according to the identified clusters, defining basic kinetic response patterns used for classification of dynamic biomarker candidates.

The following kinetic response patterns could be defined: sustained response (basically constant concentration over time, overlaid with biological and instrumental noise), early response (significant change in concentration at the beginning of exercise), late response (continuous decrease/increase towards the end of activity), and delayed response (first basic sustained response, with a strong response and steep decrease/increase in concentration after the end of the exercise during the recovery phase).

The selected two-step data analysis and modeling strategy including MFCs in concentrations and statistical hypothesis testing, and the modeling of kinetic shape templates led to the identification and classification of dynamic metabolic biomarker candidates for profiling physical activity. The highest values for MFCs and *P*-values in the analyzed set of metabolites were shown for acetylcarnitine (C2) (MFC = 1.97, *P* < 0.001), yielding a late response pattern, and being classified as strong predictor and late marker. Alanine showed the highest values in the class of amino acids (MFC = 1.42, *P* < 0.001) and yielded a late response pattern, being classified as strong predictor and late marker. The only considered sugar, glucose, yet playing a key role in physical activity, yielded a delayed response pattern classified as moderate predictor (MFC = 1.32, P < 0.001) and delayed marker.

In terms of biochemical interpretation, findings were verified and interpreted according to their function in metabolic pathways, associated primarily with physical exercise (β-oxidation of fatty acids, glycolysis, and glycogenolysis). Interestingly, biomarker candidates, identified with the highest predictive value, yielded late response patterns. This might be seen in the context that lactate (also a key indicator for profiling physical activity) first shows an almost sustained response pattern before yielding an exponential increase in concentration up to a maximum physical load. The primary occurrence of late response patterns can be interpreted as a consequence of evolutionary developed regulatory mechanisms in metabolism to keep the individual's metabolic system in homeostasis after external perturbations such as spontaneous physical activity.

Using our computational approach we were able to select and classify dynamic metabolic biomarker candidates and to characterize physiologically plausible metabolite kinetic patterns in physical activity, combining the strengths of statistical testing (hypothesis testing), mathematical modeling (curve fitting) and empiric data analysis (hierarchical cluster analysis).

### Methodology

Experimental limitations and confounders in the analyzed data may result from uncertainties about the nutrition of test persons before exercising (recorded in questionnaires but not objectively verifiable), varying individual motivations and consequently different levels of exertion, potential issues during sample taking (e.g., incomplete removal of sweat at the point of puncture), or from general limitations of the analytical approach based on dried blood spots [57]. It should be noted, that at least two test persons obviously consumed nutritional supplements in the form of branch-chained amino acids, influencing the measurement values of xleucine (sum of leucine and isoleucine).

With reference to the selected cohort, it should be noted that the study participants formed a heterogeneous group, i.e., they differed in their level of physical activity and status of training. Therefore, the baseline concentrations and the kinetic patterns may, to a certain extent, depend on the volunteers' differences in physical fitness, or other confounding factors such as anthropometric measures or dietary habits. Although this paper is primarily focused on the methodology for deriving kinetic patterns and not so much on the discovery of exercise-related biochemical mechanisms, the results should be seen with these limitations in mind.

In terms of data preprocessing, the presented data analysis strategy reveals strong indifference towards the handling of outliers because median concentration values are selected from rescaled and interpolated concentration curves. Cut-off values for the selection of metabolic biomarker candidates (utilizing MFCs and *P*-values) were chosen empirically by reviewing obtained results and assuming that responses, showing changes in concentration within a range of-10% to +10%, are accepted as biochemically and analytically-caused data variability. For kinetic modeling, an empirical approach (instead of applying pre-defined mathematical basic functions) based on polynomial fitting was chosen, allowing for a more physiological characterization of metabolite kinetics. Looking at the complete set of characterized metabolite kinetic signatures, the user can choose an appropriate polynomial degree after visual inspection or by developing a proper statistical quality measure e.g., based on an estimation of the residuals. In a few cases, minor artifacts of approx. 3% in concentration values of the fitted polynomials do occur, obviously resulting from a slight overfitting of curves due to the chosen polynomial degree.

Identification of groups of similarly behaving metabolites by hierarchical cluster analysis is somewhat affected by the number of interpolated points in the concentration curves and by the degree chosen for fitted polynomials. A higher number of interpolation points as well as different degrees of polynomials were tested, showing high stability in cluster analysis, however, at a lower node height of the dendrogram the arrangement of single metabolites changes slightly between the clusters. Note that the selection of clusters basically depends on the chosen height (cut-off) of the hierarchical tree. Depending on the selected cut-off value, two metabolites, i.e., methylmalonylcarnitine (C3-DC-M) and hydroxyvalerylcarnitine (C5-OH) might also be classified as additional biomarker candidates, interesting for further investigation. Specification of kinetic shape templates finally builds upon the number of specified clusters, depending computationally on the selected cut-off in hierarchical cluster analysis and biochemically on the eligibility and meaningfulness of clustered templates in terms of metabolite kinetics.

### Previous work

Metabolic concentration data used in this study have served as a database for the development and validation of novel data mining and biomarker discovery strategies in previously published studies by our group. In Netzer *et al*., 2011 [58] we presented a two-step network-based approach for the identification of metabolic biomarkers, classifying alanine, acetylcarnitine (C2), propionylcarnitine (C3), and glycine, as strong, and arginine, citrulline, and lysine as moderate biomarker candidates, represented as major hubs in the dynamic network. These findings show high accordance with identified dynamic metabolic biomarker candidates in physical activity using the approach presented in this work, again selecting alanine, acetylcarnitine (C2), propionylcarnitine (C3) as strong predictors, and arginine as moderate marker candidate.

In a second paper [59] we introduced a method for biomarker identification by inferring two different types of networks, i.e., correlation networks and ratio networks. This more theoretical approach calculates scores to prioritize features using topological descriptors. Groups of obese test persons (with a body mass index (BMI) > 30) and healthy controls were compared in this study, which identified highly discriminatory biomarker candidates, i.e., histidine, ornithine, acetylcarnitine (C2), and proline.

### Conclusions

In this article, we have presented a computational methodology for dynamic biomarker classification and modeling of kinetic metabolic patterns in physical activity. Insight into kinetic regulatory mechanisms could be provided by characterizing specific kinetic signatures for selected key metabolites within the groups of acylcarnitines and amino acids, and for glucose. A new data analysis strategy for the characterization and classification of dynamic biomarker candidates was introduced. We were able to specify common kinetic shape templates, identified from groups of metabolites showing a similar characteristic in dynamic time-course responses. Findings demonstrated high accordance with previously published data and established biochemical knowledge, e.g., the response of glucose, showing a behavior similar to a hockey stick function with a delayed increase in concentration after the end of physical exercise during the recovery phase.

Due to the selected study design of a cycle ergometry experiment, in which physical exercise was increased incrementally (every 3 minutes by 25 W), known kinetic patterns could be partly confirmed by our observations, in particular in response to the selected workload protocol. Major impact of the presented methodology can be seen in the fact that kinetic mechanisms in metabolism could be qualified and quantified not only through a “strong” mathematical model, but by an empiric deduction and description of *de facto* kinetic response patterns from quantitated metabolic time-course concentration data, measured under *in-vivo* experimental conditions.

A further direction of research might be the analysis of additional classes of metabolites and the description and interpretation of kinetic patterns subsequent to active exercise in the recovery phase. Especially for glucose—which increases rapidly in concentration within the analyzed interval of the recovery phase—a prolonged examination time would be highly interesting, since glucose might be expected to be classified as strong predictor. From the computational viewpoint, a very challenging task would be the development of *in-silico* pathway models, integrating the identified kinetic signatures into a theoretical mathematical model for hypothesis generation and verification (see e.g., Teusink *et al*., 2000 [60]). The development of a kinetic model based on an ordinary differential equations (ODEs) description including kinetic parameters selected from our research might be an aim for additional research which, however, is beyond the scope of this article.

The approach presented in this work also shows high potential for contributing to other application areas such as pathophysiology and pharmacodynamics. In pharmacodynamics and toxicology (particularly in chronic toxicity testing), for instance, it might be applied to assess treatment effects more accurately by profiling metabolite levels over time instead of looking at end-points only (see [29]). In many complex diseases, the dynamic analysis may well identify more meaningful biomarkers and reveal a deeper insight into the actual pathomechanisms. To name one important example that is actually very close to the present study: in chronic obstructive pulmonary disease (COPD), physical exercise—and bicycle ergometry in particular—is commonly used to assess the severity of the disease and also to model exacerbations of the patients’ condition [61,62]. In this setting, a dynamic depiction of the metabolic changes clearly has the potential to resolve regulatory mechanisms and distinguish cause and effect of the observed alterations (Christian Schudt, personal communication). This is especially plausible for the pathway leading to the synthesis of inflammation mediators such as prostaglandins, leukotrienes, thromboxanes etc., which is closely associated with the pathology of the disease and depends on the release of polyunsaturated fatty acids from phospholipids in a stoichiometric manner [63–66].

In this article, main focus was put on the development of a computational methodology to examine longitudinal metabolic concentration data and to present a basic approach for the mathematical modeling and statistical analysis of dynamic kinetic metabolic mechanisms. As previously stated (see section Methodology), individual metabolic response patterns are partly influenced by different factors such as physical fitness and training status, anthropometric parameters or dietary habits. Because of limitations in the specification and verification of the observed metabolic kinetic patterns, a further research goal might be to systematically investigate the underlying metabolic and physiological regulatory mechanisms by conducting additional hypothesis-driven prospective cohort studies. Furthermore, an extension of this paper is planned that will compare specific groups of interest, e.g., defined with regard to training status (response in lactate increases) or anthropometric characteristics.

Referring to the practical execution of exercise physiology experiments, it should be noted that most commonly only one blood sample is collected, usually after the end of exercise. However, the results of the presented work clearly demonstrate that characterized metabolites show a very differential kinetic characteristic during physical activity. Consequently one-point measurements may lead to misinterpretations and emphasize an obvious need for multiple measurements in exercise physiology (typically before, multiple times during, and after exercise).

## Materials and Methods

### Ethics statement

This study was conducted in full accordance with the principles expressed in the Declaration of Helsinki. Written informed consent was obtained from all study participants, together with a detailed questionnaire on nutrition and health status. In addition, a physician subjected all individuals to a detailed examination to ensure that they could undergo the cycle ergometry test without health risks, and this physician was also present at all times during the exercise to monitor the electrocardiogram (ECG) that was continuously recorded. All laboratory work and data analysis was conducted anonymously.

### Experimental study design

In this work, longitudinal metabolic concentration data were obtained through clinical exercise testing using a cycle ergometry stress test. General guidance for clinical exercise testing can be found in "Guidelines for Clinical Exercise Testing Laboratories" [67] and "Recommendations for Clinical Exercise Laboratories" [68]. General recommendations for cycle ergometry studies were described by Driss & Vandewalle, 2013 [69], providing technical and clinical protocols including limitations for study design and execution. The overall cycle ergometry experiment was designed as a longitudinal biomarker cohort study, with 47 persons divided into 4 different groups, i.e., male and female individuals, with either average physical activity or competitive athletic activity. Study participants included amateur endurance athletes (16 males / 8 females) and professional alpine skiers (11 males / 12 females). The anthropometric characteristics of the study participants (age, body mass index (BMI), height, and weight) are summarized in Table 3. Detailed information on anthropometric data, the general training status, and the physical load during the cycle ergometer experiment are provided as supporting information (S3 File).

**Table 3:** Anthropometric data.

Measure	Mean	Minimum	Maximum
Age [y]	34.19	20	53
Body mass index (BMI)	23.27	18.42	28.98
Height [m]	1.75	1.60	1.86
Weight [kg]	71.35	52	96

Summary of anthropometric data of study participants. Mean, minimum and maximum values are denoted for age, body mass index (BMI), height and weight.

### Clinical study execution

The workload of the cycle ergometry test was increased incrementally by 25 W every 3 minutes up to the individual’s maximum physical load (the basic scheme of the study protocol is depicted in S1 Fig). The initial workload level of 25 W was skipped for all individuals, starting the exercise with a workload of 50 W. The lowest observed maximum workload was 150 W (one individual), and the highest workload level was 425 W, also reached by one individual. From each individual blood samples for metabolite profiling were taken (i) at rest (directly before starting the exercise), (ii) with each new workload level up to individual’s maximum physical load, and (iii) after a short recovery phase of five minutes after the maximum workload (highest Watt level). In addition, for all test subjects, lactate concentrations were measured as a gold standard and reference for assessing physical activity. Concentration-time curves of preprocessed lactate data are visualized in S2 Fig. Median values of lactate concentrations were roughly 1.2 mM at rest, 8.5 mM at maximum workload, and 7.2 mM after recovery. According to the study protocol, lactate samples were taken at 1:30 min, samples for metabolite profiling at 2:30 min after starting a new ergometry workload level. All samples were taken from the earlobe, collected as dried blood spots (DBS) [57] and analyzed under standardized study conditions.

### Targeted metabolite quantitation

In metabolomics, two basic conceptual approaches are used: untargeted and targeted metabolite profiling methods. Untargeted metabolomics seeks to create a holistic picture of metabolism by trying to identify a comprehensive set of metabolites as a snapshot of a metabolic state, while targeted metabolomics aims at a quantitation of pre-selected metabolites defined by *a priori* knowledge [70,71]. The two state-of-the-art technologies for analyzing metabolites are nuclear magnetic resonance (NMR) spectroscopy [72] and mass spectrometry (MS) [73]. Dynamic time-course metabolic concentration values, building the basis for data analysis and modeling in this work, were gathered from a targeted metabolomics approach [70,74,75], using triple quadrupole tandem mass spectrometry (MS/MS) [76] coupled with the concept of stable isotope dilution (SID) [77] for metabolite quantitation.

### Measured metabolite concentrations

Longitudinal metabolite concentration data were quantified for three classes of metabolites: acylcarnitines, amino acids, and sugars. In total, 110 metabolites were measured: 40 acylcarnitines, 18 amino acids, and 52 sugars. Quantitated concentration data of all measured metabolites were thoroughly examined with respect to data quality assurance and reliability. Metabolites either below the detection limit (LOD) of 50 nM, measurements with lots of missing values or wide variabilities were excluded from this analysis. As result, targeted concentration data of a selected set of 30 metabolites are considered for data analysis in this work: 11 acylcarnitines, 18 amino acids, and 1 sugar. Analyzed acylcarnitines include: carnitine (C0), acetylcarnitine (C2), propionylcarnitine (C3), methylmalonylcarnitine (C3-DC-M), butyrylcarnitine (C4), valerylcarnitine (C5), hydroxyvalerylcarnitine (C5-OH), hexadecanoylcarnitine (C16), octadecanoylcarnitine (C18), octadecenoylcarnitine (C18:1), and octadecadienylcarnitine (C18:2). Amino acids are: alanine, arginine, aspartic acid, citrulline, glutamic acid, glycine, histidine, lysine, methionine, ornithine, phenylalanine, proline, serine, threonine, tryptophan, tyrosine, valine, and xleucine (the sum of leucine and isoleucine). Analyzed metabolite within the class of sugars was glucose. Collected data were almost complete, except some missing data at individual’s maximum load (twelve individuals, however lactate could be measured after 1:30 min for all of them), and at 150 W for one test person (no. 9).

### Data analysis workflow

**Fig 6:**
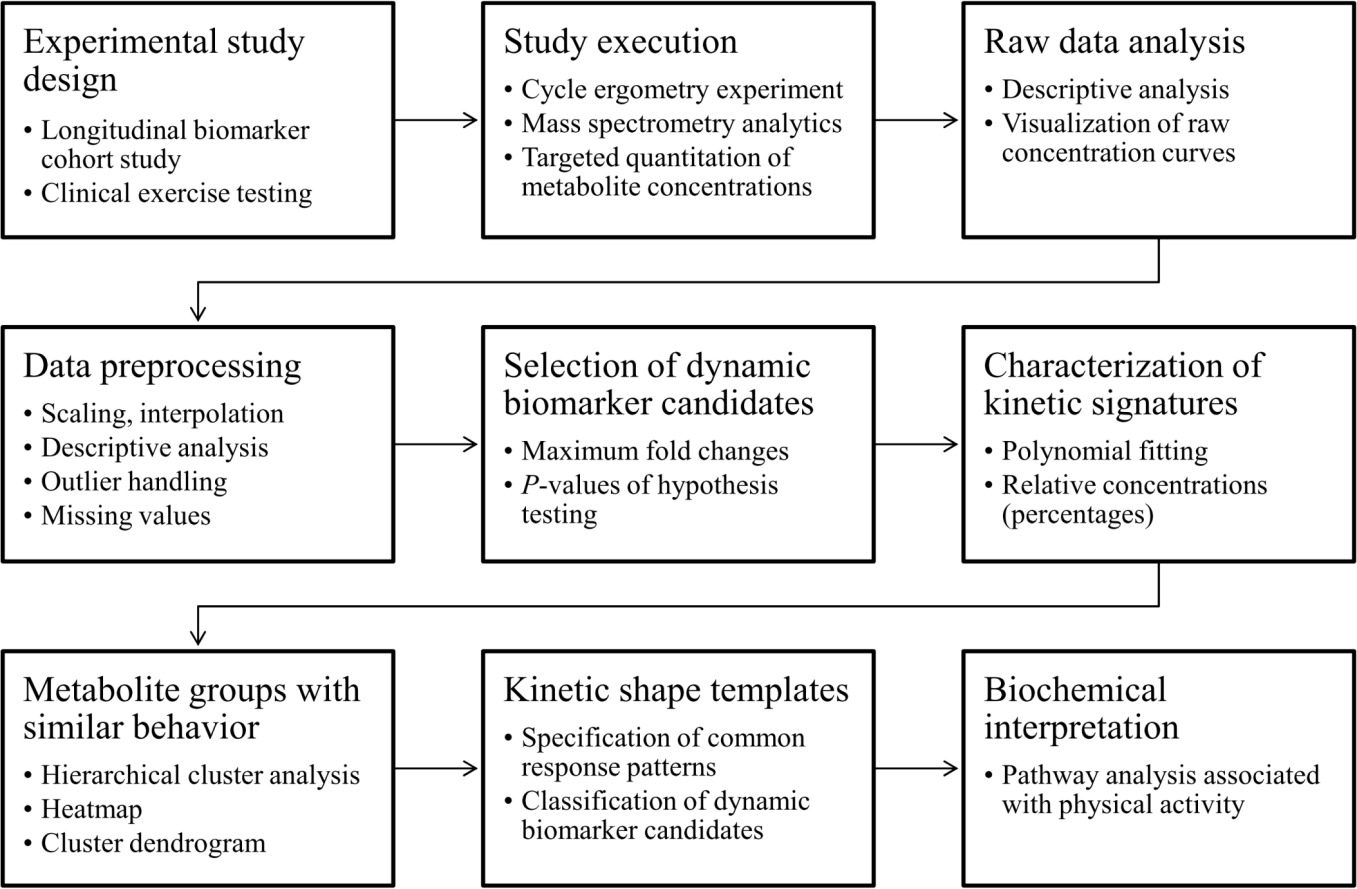
Data analysis workflow. Flow chart of the selected data analysis and biomarker discovery workflow (according to the workflow described by Baumgartner & Graber, 2008 [42]). Intermediate discovery steps include the technical validation of raw data, preprocessing of data, selection of dynamic biomarker candidates, modeling and characterization of metabolite kinetic patterns, identification of metabolite groups with similar kinetic behavior, specification of observed kinetic shape templates, classification of dynamic biomarker candidates, and subsequently the biochemical interpretation of findings.

Central steps of the selected data analysis workflow include the technical validation of raw data, preprocessing of data, selection of putative dynamic biomarker candidates, mathematical modeling and characterization of metabolite kinetic patterns, identification of metabolite groups with similar kinetic behavior, specification of observed kinetic shape templates, classification of dynamic biomarker candidates and the biochemical interpretation of findings. A flowchart of the used data analysis workflow is shown in Fig 6, representing the whole data-driven process for the discovery of biomarkers in metabolomics. Results from the different steps of the data analysis workflow are exemplarily shown and visualized for glucose, a key analyte, playing a central role in metabolism of exercise physiology and demonstrating a very specific kinetic pattern in response to physical activity.

### Raw data—descriptive analysis and visualization

Raw data of the cycle ergometry experiment were test-wise reviewed and visualized in two different basic ways to obtain a better understanding about the nature of the metabolic time-course data. Concentration data were initially analyzed by building subsets of data, referring to the levels of each individual’s maximum physical load. For each subset a box plot was generated, visualizing the specific measurements (data points of the horizontal axis) versus the metabolite concentrations (see section Clinical study execution). In S3 Fig, resulting box plots for glucose are exemplarily shown. Eight box plots were generated, where the lowest value of individual maximum workload (150 W) resulted in 7 data points (1 test person) and the highest value (425 W) in 18 measurement points (1 test person). Second, analyzed metabolites were visualized as raw concentration curves (exemplarily shown for glucose in S4 Fig), illustrating differences in individual workload and time of exercise of examined test persons. The horizontal axis hereby represents time points of measurements in minutes.

### Data preprocessing

**Fig 7:**
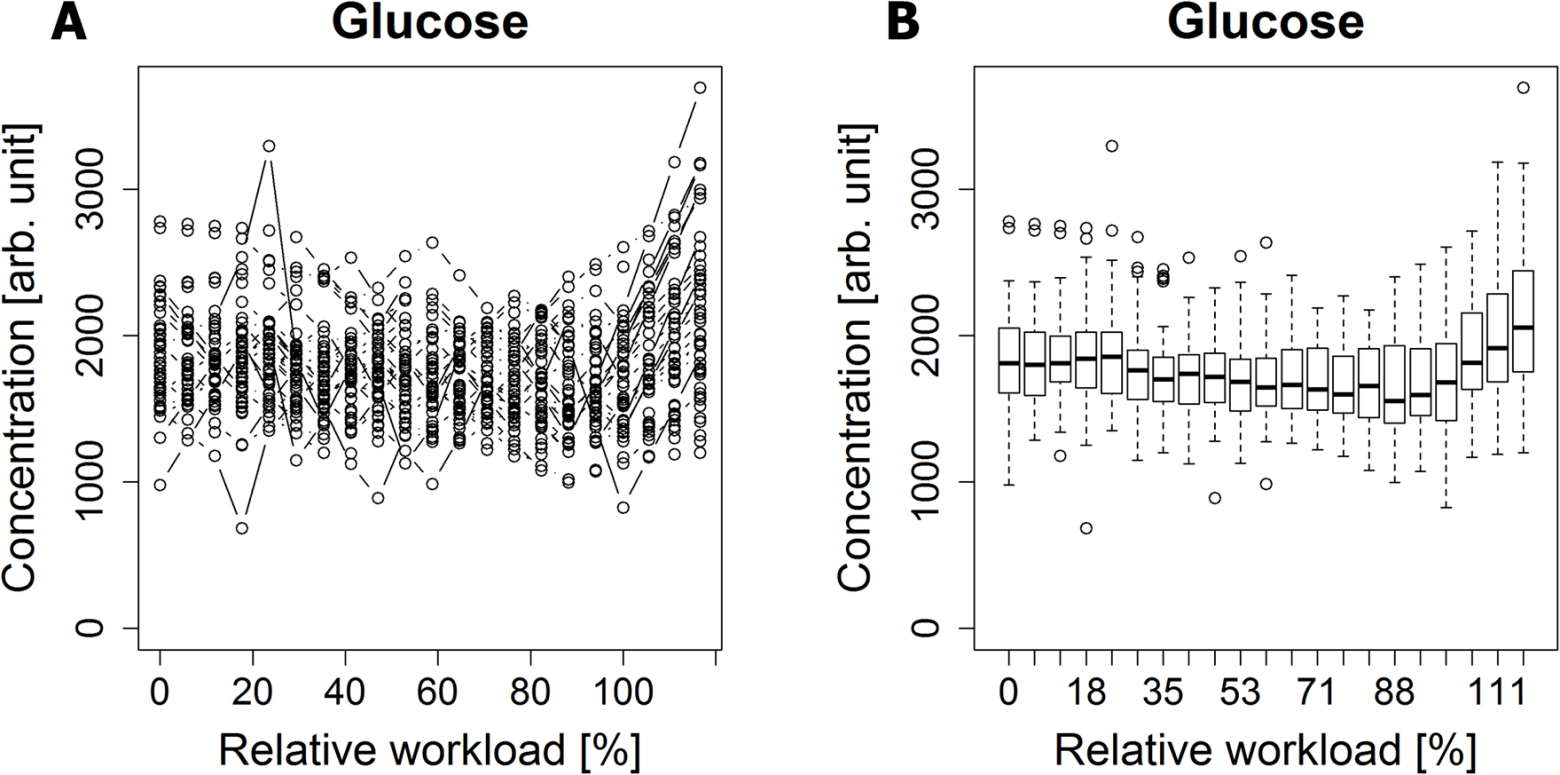
Glucose concentration curves. A) Concentration curves of all test persons after linear interpolation. B) Box plot representation of concentration curves of all test persons.

Different lengths of metabolic concentration-time curves, resulting from the variability of each individual’s maximum physical load, were made comparable by rescaling the data in time (S5 Fig). Measurement at rest was defined as 0%, maximum workload of each individual as 100% and recovery value as median value of 117%—resulting in an aligned workload curve to a uniform length. This requires additional data points added to the concentration curves using a linear interpolation approach (Fig 7A).

Metabolic concentration-time curves underwent simple descriptive analysis by generating a box plot representation from rescaled concentration curves (Fig 7B). In a next step, median concentration values were extracted from interpolated concentration curves (S6 Fig), serving as a basis for mathematical modeling by curve fitting (see section Mathematical modeling). This approach perfectly treats the problem of outliers in the data without the need of applying additional methods for outlier detection and removal. However, a small set of extreme outliers was observed that was manually removed after careful visual inspection (in test person no. 14 all data points at recovery, in test person no. 35 all data points at 175 W and in test person no. 42 the data point for glucose at rest). Regarding missing concentration values it should be noted that our dataset was almost complete, except missing values at individuals’ maximum workload in 12 test persons and at 150 W in one individual (no. 9), representing the last data points in the concentration time curves.

### Maximum fold changes and statistical hypothesis testing

Maximum fold changes in metabolite concentrations and *P*-values of statistical hypothesis testing serve as a score for the ranking of putative biomarker candidates (see section Selection of dynamic biomarker candidates). The combination of fold changes and *P*-values, visualized using a volcano plot, is described in the literature as method of choice for the analysis and visualization of significant changes (e.g., on microarray data [78,79] or in diverse metabolomics applications [80–82]). As a general approach, especially in genomics studies, this method is usually used for data comparing the starting and end point of dynamic processes such as regulation of gene expression.

In this work, utilizing longitudinal time course concentration data, MFCs are calculated by the difference between observed minimum and maximum concentration values of a metabolite, independently from their timely occurrence. MFCs are calculated based on median concentration values extracted from interpolated concentration curves (see section Data preprocessing). Measurement indices are determined, and consequently, if the minimum concentration occurs earlier in the time course, the ratio of maximum concentration to minimum concentration is calculated and vice versa. This modality is summarized in the following pseudo-code:

*if (conc_min_index < conc_max_index)*

maximum_fold_change=conc_max/conc_min (5)

*else*

maximum_fold_change=conc_min/conc_max (6)

*P*-values of statistical hypothesis testing are calculated in a similar way, first by determining measurement index positions of minimum and maximum median concentration values of interpolated concentration curves and in a subsequent step, by extracting interpolated individual concentration values at identified index positions as basis for statistical hypothesis testing. Interpolated minimum and maximum concentration values of all 30 metabolites were assessed with respect to their density distribution by visual inspection using graphical methods such as histogram analysis [83] and quantile-quantile plots [84]. A Shapiro-Wilk Normality test was applied for normality testing of both minimum and maximum concentration data (significance level *P* = 0.01) [85–87]. Metabolites hereby yielded inhomogeneous distributions (e.g., normal distribution for histidine, octadecanoylcarnitine (C18) or glycine, non-normal distribution e.g., for xLeucine, citrulline or proline, and partly differences in distributions between minimum and maximum concentrations, e.g., for arginine). To ensure comparability between metabolites, a Wilcoxon Signed Rank Test [88] (non-parametric hypothesis test for paired samples) was used for the calculation of *P*-values (significance level *P* = 0.001). Since ranks are used for paired hypothesis testing, identical *P*-values are partly shown for some metabolites. Finally, calculated *P*-values were adjusted according to the false discovery rate (FDR) correction for multiple comparisons [89].

### Mathematical modeling

The initial goal of our work was the mathematical modeling of metabolite kinetic patterns and shape templates, utilizing a set of predefined mathematical basis functions [29]. However, the introduction of predefined basic functions for the analysis of dynamic metabolomics data is and remains a challenge as also discussed by Smilde *et al*., 2010 [29]. Note that putative basic functions in this work are associated with kinetic patterns in response to linear increasing physical activity and can be basically classified into the following set of shape templates:

- a sustained response pattern, showing a mainly constant concentration over time, overlaid with biological or instrument noise, represented e.g., by a constant function
  f(x)=c (7)
- an early response pattern, with an early significant change in concentration, mathematically described e.g., by a logarithmic function
  f(x)=ln(x) (8)
- a halving interval response, showing a change in concentration at the half time of exercise with a following plateau, represented e.g., by a sigmoid basis function
  $$f\left(x\right)=\frac{1}{\left(1+e^{-t}\right)}$$ (9)
- a late response, with a continuous decrease/increase up to the end of physical activity, described e.g., by a linear, a quadratic, or an exponential function
  f(x)=cx (10)
  $$f\left(x\right)=x^{2}$$ (11)
  $$f\left(x\right)=e^{x}$$ (12)
- a delayed response pattern, showing first a sustained characteristic followed by a strong response after the end of physical exercise during the recovery phase, mathematically represented by a so-called L-curve or hockey stick function [90,91].

Fitting the above-introduced basic functions to measured concentration-time curves was thoroughly examined and tested with the goal to characterize kinetic response patterns according to these theoretical models. In this analysis curve fitting was performed using median metabolite concentration values, extracted from interpolated concentration-time curves (see section Data preprocessing). Our preliminary results demonstrated that the approach of fitting the pre-defined set of mathematical basis functions was not feasible for the measured response curves caused by an incremental increase of physical workload. We therefore revised our initial concept by utilizing polynomial fitting of preprocessed data. This modality enables us to design kinetic response patterns that are physiologically reasonable and relevant. Polynomials of degree *n* are defined by following equation:

$$f\left(x\right)=\sum_{i=0}^{n}\;a_{i}x^{i}$$ (13)

After testing different polynomial degrees, we decided for a degree of nine, showing the best results in terms of curve/shape representation and smoothness (S7 Fig). To ensure comparability of analyzed metabolites after polynomial fitting, relative concentration values were calculated (in percentage of concentration changes with respect to the initial concentration at rest) (see Fig 4).

Note that there are multiple applications in metabolomics using polynomial fitting, e.g. for baseline correction [92,93], prediction of germination curves [94], calculation of mass correction profiles [95] or in spectrum deconvolution [96].

### Hierarchical cluster analysis

Metabolite groups, showing similar kinetic response patterns, were examined and identified using hierarchical cluster analysis [97]. Cluster analysis was performed based on the concentration values of the fitted polynomials of 9^th^ degree (see section Mathematical modeling).

**Fig 8:**
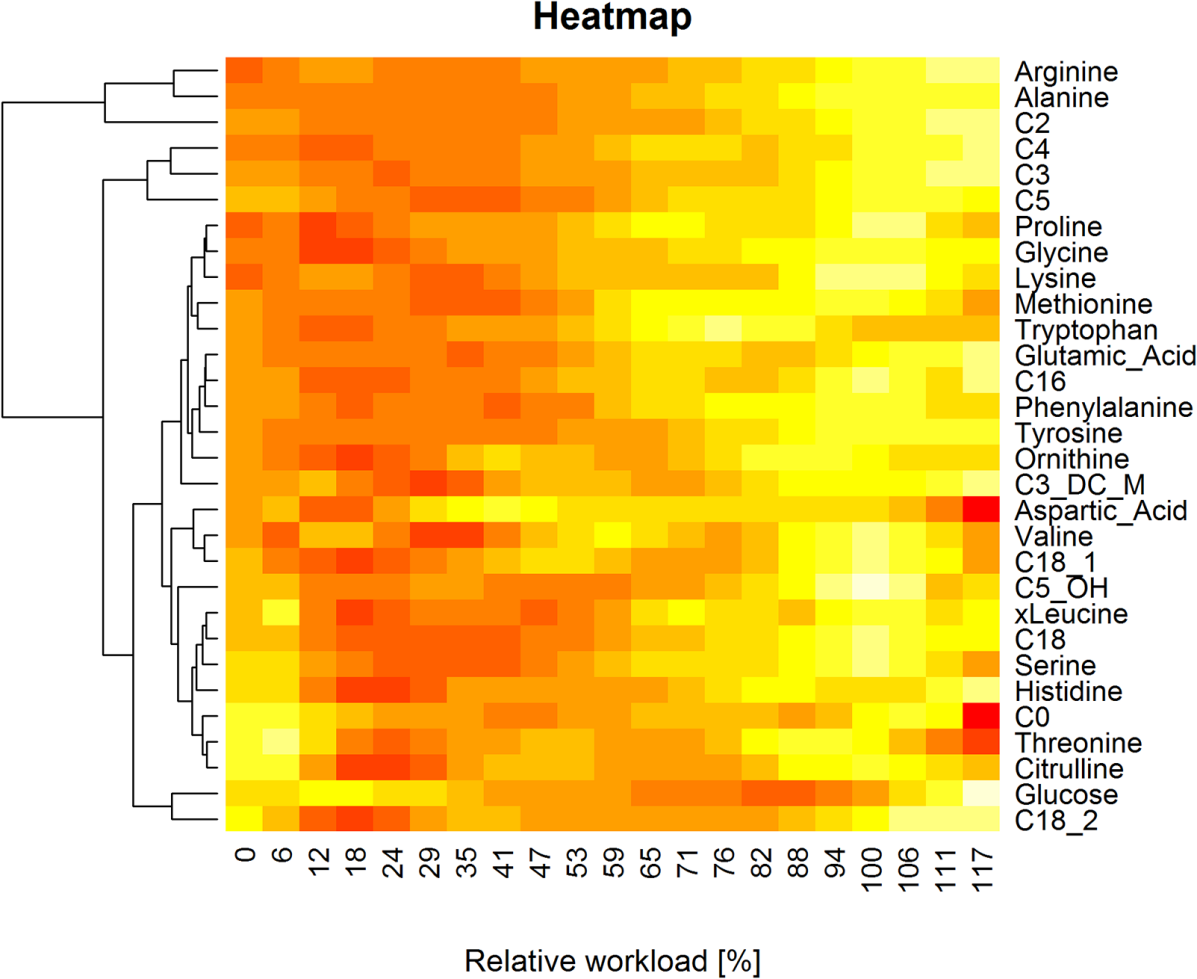
Heatmap. Colored heatmap, visualizing the results of hierarchical cluster analysis. Concentration values are scaled and centered for each metabolite by row, resulting in an improved color representation. Relative workload values (x-axis) are visualized in linear order, resulting in a colored representation of the polynomially fitted concentration curves for each metabolite.

Results are visualized as a heatmap in Fig 8. Relative workload values of the x-axis are displayed in linear order. Red colors indicate lower values in dynamic time-course concentrations of specific metabolites, lighter colors indicate higher concentration values. The resulting cluster dendrogram is separately shown in S8 Fig. Clusters of metabolites, showing similar dynamic behavior, were empirically identified by cutting the hierarchical tree at a threshold of 35, resulting in seven different metabolite clusters (see section Identification of metabolite groups with similar patterns).

Descriptive statistical analysis for each cluster was performed and corresponding box plots were generated (S9 Fig). Median concentration curves of each cluster, allowing for an accurate representation and specification of kinetic shape templates, were selected (S10 Fig) (see section Specification of kinetic shape templates).

## Supporting Information

S1 Fig Study protocol. Study protocol of the cycle ergometry performance test, exemplarily shown for the maximum workload level of 425 W. Workload is increased by 25 W every 3 minutes (25 W level is skipped). Blood samples are taken at rest, with every new workload level, and after a recovery period of five minutes after the individual maximum workload.
(TIF)

S2 Fig Lactate concentration curves. Lactate concentration curves of the 47 analyzed individuals. To ensure comparability of individual workload curves, concentration curves are rescaled and values interpolated (see section Data preprocessing).
(TIF)

S3 Fig Raw data box plots. Box plots for descriptive analysis of raw data. Data are visualized by grouping persons into subgroups reaching the same maximal workload level. The minimum number of measurements is seven (at 150 W of individual’s maximum load) and the maximum number of measurements is 18 (at 425 W).
(TIF)

S4 Fig Glucose raw concentration curves. Visualization of raw concentration curves for glucose, where data are visualized with regard to time points of measurement. The shortest individual workload curve ends after approx. 23 minutes, the longest individual workload curve after approx. 56 minutes.
(TIF)

S5 Fig Glucose rescaled concentration curves. To ensure comparability of differing individual workload curves, a rescaling of data is performed referring to the individual maximum physical load as 100%. The values for recovery are rescaled with respect to the median recovery value of 117%.
(TIF)

S6 Fig Glucose median concentration values. Glucose median concentration values extracted from interpolated concentration curves, which are used as a basis for mathematical modeling.
(TIF)

S7 Fig Glucose fitted polynomial. Mathematical modeling by fitting a polynomial of 9^th^ degree to median concentration values, extracted from interpolated concentration values.
(TIF)

S8 Fig Hierarchical cluster dendrogram. Dendrogram of hierarchical clusters analysis. Metabolites acetylcarnitine (C2), valerylcarnitine (C5), propionylcarnitine (C3), butyrylcarnitine (C4), octadecadienylcarnitine (C18:2), glucose, alanine, and arginine are separated at a high cut-off level.
(TIF)

S9 Fig Cluster box plots. For each cluster a box plot for a descriptive representation is generated. Median values are selected from the concentration curves of each cluster, serving as basis for the specification of kinetic shape templates.
(TIF)

S10 Fig Cluster median concentration curves. Median concentration curves of all metabolite clusters (identified through hierarchical cluster analysis), serving as basis for the specification of kinetic shape templates.
(TIF)

S1 File Source file. R script file for the modeling of kinetic patterns of dynamic metabolic biomarkers.
(ZIP)

S2 File Underlying dataset. Microsoft Excel file containing longitudinal metabolic concentration data.
(ZIP)

S3 File Participant characteristics. Anthropometric data and workload performed on the cycle ergometer, listed for each study participant.
(ZIP)

## References

[1] Guldberg CM, Waage P. Studier i affiniteten. (Translation: Studies on affinities.) Forhandlinger i Videnskabs-Selskabet i Christiania 1864.

[2] Guldberg CM, Waage P. E´tudes sur les affinites chimiques. (Translation: Studies on chemical affinities.) Christiania: Brøgger & Christie 1867.

[3] Guldberg CM, Waage P. Über die chemische Affinität. (Translation: On chemical affinity.) Erdmann’s Journal für practische Cehmie. 1879;127:69–114.

[4] Voit E, Martens H, Omholt SW. 150 years of the mass action law. PLoS Comput Biol. 2015;11(1):e1004012 10.1371/journal.pcbi.1004012 25569257 PMC4288704

[5] Michaelis L, Menten ML. Die Kinetik der Invertinwirkung. (Translation: The kinetics of invertase activity.) Biochemische Zeitschrift. 1913;49:333–369.

[6] Devlin TM (Ed.). Textbook of Biochemistry with Clinical Correlations 6th ed. Hoboken, NJ: Wiley-Liss; 2006.

[7] Nelson DL, Cox MM. Lehninger Principles of Biochemistry 5th ed. New York: W. H. Freeman and Company; 2008.

[8] Koch D, Lu C, Fisker-Andersen J, Jolley L, Fenske RA. Temporal association of children's pesticide exposure and agricultural spraying: report of a longitudinal biological monitoring study. Environmental health perspectives. 2002;110(8):829 12153767 10.1289/ehp.02110829 PMC1240957

[9] Lu C, Barr DB, Pearson MA, Waller LA. Dietary intake and its contribution to longitudinal organophosphorus pesticide exposure in urban/suburban children. Environmental health perspectives. 2008;116(4):537 10.1289/ehp.10912 18414640 PMC2290988

[10] Pomponio G, Zurich MG, Schultz L, Weiss DG, Romanelli L, Gramowski-Voss A, et al Amiodarone biokinetics, the formation of its major oxidative metabolite and neurotoxicity after acute and repeated exposure of brain cell cultures. Toxicol In Vitro. 2015. pii:S0887–2333(15)00013–2. 10.1016/j.tiv.2015.01.012 25659768

[11] Barmentlo SH, Stel JM, van Doorn M, Eschauzier C, de Voogt P, Kraak MH. Acute and chronic toxicity of short chained perfluoroalkyl substances to Daphnia magna. Environ Pollut. 2015;198:47–53. 10.1016/j.envpol.2014.12.025 25553346

[12] Mackevica A, Skjolding LM, Gergs A, Palmqvist A, Baun A. Chronic toxicity of silver nanoparticles to Daphnia magna under different feeding conditions. Aquat Toxicol. 2015;161C:10–16. 10.1016/j.aquatox.2015.01.023 25661705

[13] Yang S, Giannone RJ, Dice L, Yang ZK, Engle NL, Tschaplinski TJ, et al Clostridium thermocellum ATCC27405 transcriptomic, metabolomic and proteomic profiles after ethanol stress. BMC Genomics. 2012;13:336 10.1186/1471-2164-13-336 22823947 PMC3478167

[14] Castro CC, Martins RC, Teixeira JA, Silva Ferreira AC. Application of a high-throughput process analytical technology metabolomics pipeline to Port wine forced ageing process. Food Chem. 2014;143:384–91. 10.1016/j.foodchem.2013.07.138 24054256

[15] Chong WP, Yusufi FN, Lee DY, Reddy SG, Wong NS, Heng CK, et al Metabolomics-based identification of apoptosis-inducing metabolites in recombinant fed-batch CHO culture media. J Biotechnol. 2011;151(2):218–24. 10.1016/j.jbiotec.2010.12.010 21167884

[16] Selvarasu S, Ho YS, Chong WP, Wong NS, Yusufi FN, Lee YY, et al Combined in silico modeling and metabolomics analysis to characterize fed-batch CHO cell culture. Biotechnol Bioeng. 2012;109(6):1415–29. 10.1002/bit.24445 22252269

[17] Mohmad-Saberi SE, Hashim YZ, Mel M, Amid A, Ahmad-Raus R, Packeer-Mohamed V. Metabolomics profiling of extracellular metabolites in CHO-K1 cells cultured in different types of growth media. Cytotechnology. 2013;65(4):577–86. 10.1007/s10616-012-9508-4 23179090 PMC3720965

[18] Cao B, Li M, Zha W, Zhao Q, Gu R, Liu L, et al Metabolomic approach to evaluating adriamycin pharmacodynamics and resistance in breast cancer cells. Metabolomics. 2013;9(5):960–973. 24039617 10.1007/s11306-013-0517-x PMC3769585

[19] Chen Y, Ren J, Qin X, Li G, Zhou B, Gu G, et al Metabolism of Albumin after Continuous Venovenous Hemofiltration in Patients with Systemic Inflammatory Response Syndrome. BioMed Research International. 2015;2015:917674 10.1155/2015/917674 25650044 PMC4310232

[20] Campbell C, Grapov D, Fiehn O, Chandler CJ, Burnett DJ, Souza EC, et al Improved metabolic health alters host metabolism in parallel with changes in systemic xeno-metabolites of gut origin. PLOS ONE. 2014;9(1):e84260 10.1371/journal.pone.0084260 24416208 PMC3885560

[21] Knudsen SH, Karstoft K, Pedersen BK, van Hall G, Solomon TP. The immediate effects of a single bout of aerobic exercise on oral glucose tolerance across the glucose tolerance continuum. Physiol Rep. 2014;2(8):e12114 10.14814/phy2.12114 25168869 PMC4246585

[22] Rios-Estepa R, Lange BM. Experimental and mathematical approaches to modeling plant metabolic networks. Phytochemistry. 2007;68(16):2351–2374. 17561179 10.1016/j.phytochem.2007.04.021

[23] Steuer R, Junker BH. Computational models of metabolism: stability and regulation in metabolic networks In: Rice SA (Ed.). Advances in chemical physics, vol. 142 Hoboken, NJ: John Wiley & Sons; 2009.

[24] Heijnen JJ. Approximative kinetic formats used in metabolic network modeling. Biotechnology and bioengineering. 2005;91(5):534–545. 16003779 10.1002/bit.20558

[25] Modre-Osprian R, Osprian I, Tilg B, Schreier G, Weinberger KM, Graber A. Dynamic simulations on the mitochondrial fatty acid beta-oxidation network. BMC Syst Biol. 2009;3:2 10.1186/1752-0509-3-2 19126203 PMC2633313

[26] Takahashi K, Ishikawa N, Sadamoto Y, Sasamoto H, Ohta S, Shiozawa A, Miyoshi F, Naito Y, Nakayama Y, Tomita M. E-Cell 2: multi-platform E-Cell simulation system. Bioinformatics. 2003;19(13):1727–9. 15593410 10.1093/bioinformatics/btg221

[27] Smolen P, Hardin PE, Lo BS, Baxter DA, Byrne JH. Simulation of Drosophila Circadian Oscillations, Mutations, and Light Responses by a Model with VRI, PDP-1, and CLK. Biophysical journal. 2004;86(5):2786–2802. 15111397 10.1016/S0006-3495(04)74332-5 PMC1304149

[28] Nishino T, Yachie-Kinoshita A, Hirayama A, Soga T, Suematsu M, Tomita M. Dynamic simulation and metabolome analysis of long-term erythrocyte storage in adenine-guanosine solution. PLoS ONE. 2013;8(8):e71060 10.1371/journal.pone.0071060 24205395 PMC3796775

[29] Smilde AK, Westerhuis JA, Hoefsloot HCJ, Bijlsma S, Rubingh CM, Vis DJ, et al Dynamic metabolomic data analysis: a tutorial review. Metabolomics. 2010;6(1):3–17. 20339444 10.1007/s11306-009-0191-1 PMC2834778

[30] Bakshi BR. Multiscale pca with application to multivariate statistical process monitoring. AIChE Journal. 1998;44:1596–1610.

[31] Schliep A, Schonhuth A, Steinhoff C. Using hidden markov models to analyze gene expression time course data. Bioinformatics. 2003;19:i255–i263. 12855468 10.1093/bioinformatics/btg1036

[32] Storey JD, Xiao W, Leek JT, Tompkins RG, Davis RW. Significance analysis of time course microarray experiments. Proceedings of the National Academy of Sciences of the United States of America. 2005;102(36):12837–12842. 16141318 10.1073/pnas.0504609102 PMC1201697

[33] Conesa A, Nueda MJ, Ferrer A, Talon M. Masigpro: A method to identify significantly differential expression profules in time-course microarray experiments. Bioinformatics. 2006;22(9):1096–1102. 16481333 10.1093/bioinformatics/btl056

[34] Jansen JJ, Hoefsloot HC, Boelens HF, Van Der Greef J, Smilde AK. Analysis of longitudinal metabolomics data. Bioinformatics. 2004;20(15):2438–2446. 15087313 10.1093/bioinformatics/bth268

[35] Smilde AK, Jansen JJ, Hoefsloot HC, Lamers RJA, Van Der Greef J, Timmerman ME. ANOVA-simultaneous component analysis (ASCA): a new tool for analyzing designed metabolomics data. Bioinformatics. 2005;21(13):3043–3048. 15890747 10.1093/bioinformatics/bti476

[36] Berk M, Ebbels T, Montana G. A statistical framework for biomarker discovery in metabolomic time course data. Bioinformatics. 2011;27(14):1979–1985. 10.1093/bioinformatics/btr289 21729866 PMC3129523

[37] Mishina EV, Straubinger RM, Pyszczynski NA, Jusko WJ. Enhancement of tissue delivery and receptor occupancy of methylprednisolone in rats by a liposomal formulation. Pharmaceutical research. 1993;10(10):1402–1410. 8272400 10.1023/a:1018954704886

[38] Jansen JJ, Szymanska E, Hoefsloot HC, Jacobs DM, Strassburg K, Smilde AK. Between Metabolite Relationships: an essential aspect of metabolic change. Metabolomics. 2012;8(3):422–432. 22661919 10.1007/s11306-011-0316-1 PMC3351608

[39] Stanberry L, Mias GI, Haynes W, Higdon R, Snyder M, Kolker E. Integrative analysis of longitudinal metabolomics data from a personal multi-omics profile. Metabolites. 2013;3(3):741–760. 10.3390/metabo3030741 24958148 PMC3901289

[40] Xia J, Mandal R, Sinelnikov IV, Broadhurst D, Wishart DS. MetaboAnalyst 2.0—a comprehensive server for metabolomic data analysis. Nucleic Acids Res. 2012;40(Web Server issue):W127–33. 10.1093/nar/gks374 22553367 PMC3394314

[41] Lundin U, Modre-Osprian R, Weinberger KM. Targeted metabolomics for clinical biomarker discovery in multifactorial diseases In: Ikehara K, editor. Advances in the Study of Genetic Disorders. Croatia: InTech; 2011 pp. 81–98.

[42] Baumgartner C, Graber A. Data mining and knowledge discovery in metabolomics In: Masseglia F, Poncelet P, Teisseire M (Eds.). Successes and new directions in data mining. London: Information Science Reference; 2008 pp. 141–166.

[43] Baumgartner C, Osl M, Netzer M, Baumgartner D. Bioinformatic-driven search for metabolic biomarkers in disease. J Clin Bioinforma. 2011;1:2 10.1186/2043-9113-1-2 21884622 PMC3143899

[44] Röschinger W, Olgemöller B, Fingerhut R, Liebl B, Roscher AA. Advances in analytical mass spectrometry to improve screening for inherited metabolic diseases. Eur J Pediatr. 2003;162 Suppl 1:S67–76. 14618396 10.1007/s00431-003-1356-y

[45] Osl M, Dreiseitl S, Pfeifer B, Weinberger K, Klocker H, Bartsch G, et al A new rule-based algorithm for identifying metabolic markers in prostate cancer using tandem mass spectrometry. Bioinformatics. 2008;24(24):2908–14. 10.1093/bioinformatics/btn506 18815183

[46] Lewis GD, Wei R, Liu E, Yang E, Shi X, Martinovic M, et al Metabolite profiling of blood from individuals undergoing planned myocardial infarction reveals early markers of myocardial injury. J Clin Invest. 2008;118:3503–3512. 10.1172/JCI35111 18769631 PMC2525696

[47] Baumgartner C, Lewis GD, Netzer M, Pfeifer B, Gerszten RE. A new data mining approach for profiling and categorizing kinetic patterns of metabolic biomarkers after myocardial injury. Bioinformatics. 2010;26(14):1745–51. 10.1093/bioinformatics/btq254 20483816 PMC2894506

[48] Altmaier E, Ramsay SL, Graber A, Mewes HW, Weinberger KM, Suhre K. Bioinformatics analysis of targeted metabolomics—uncovering old and new tales of diabetic mice under medication. Endocrinology 2008;(149):3478–3489. 18372322 10.1210/en.2007-1747

[49] Gieger C, Geistlinger L, Altmaier E, Hrabé de Angelis M, Kronenberg F, Meitinger T, et al Genetics meets metabolomics: a genome-wide association study of metabolite profiles in human serum. PLoS Genet. 2008;4(11):e1000282 10.1371/journal.pgen.1000282 19043545 PMC2581785

[50] Suhre K, Meisinger C, Döring A, Altmaier E, Belcredi P, Gieger C, et al Metabolic footprint of diabetes: a multiplatform metabolomics study in an epidemiological setting. PLoS One. 2010;5(11):e13953 10.1371/journal.pone.0013953 21085649 PMC2978704

[51] Argilés Á, Siwy J, Duranton F, Gayrard N, Dakna M, Lundin U, et al CKD273, a new proteomics classifier assessing CKD and its prognosis. PLoS One. 2013;8(5):e62837 10.1371/journal.pone.0062837 23690958 PMC3653906

[52] Duranton F, Lundin U, Gayrard N, Mischak H, Aparicio M, Mourad G, et al Plasma and urinary amino acid metabolomic profiling in patients with different levels of kidney function. Clin J Am Soc Nephrol. 2014;9(1):37–45. 10.2215/CJN.06000613 24235289 PMC3878706

[53] Nkuipou-Kenfack E, Duranton F, Gayrard N, Argilés A, Lundin U, Weinberger KM, et al Assessment of metabolomic and proteomic biomarkers in detection and prognosis of progression of renal function in chronic kidney disease. PLoS One. 2014;9(5):e96955 10.1371/journal.pone.0096955 24817014 PMC4016198

[54] Breit M, Baumgartner C, Weinberger KM. Data handling and analysis in metabolomics In: Khanmohammadi Mohammadreza (Ed.). Current Applications of Chemometrics. New York: Nova Science Publishers; 2015 pp. 181–203.

[55] Kanehisa M, Goto S. KEGG: kyoto encyclopedia of genes and genomes. Nucleic Acids Res. 2000;28(1):27–30. 10592173 10.1093/nar/28.1.27 PMC102409

[56] Ihaka R, Gentleman R. R: a language for data analysis and graphics. Journal of computational and graphical statistics. 1996;5(3):299–314.

[57] Guthrie R, Susi A. A simple phenylalanine method for detecting phenylketonuria in large populations of newborn infants. Pediatrics. 1963;32:338–43. 14063511

[58] Netzer M, Weinberger KM, Handler M, Seger M, Fang X, Kugler KG, et al Profiling the human response to physical exercise: a computational strategy for the identification and kinetic analysis of metabolic biomarkers. J Clin Bioinforma. 2011;1(1):34 10.1186/2043-9113-1-34 22182709 PMC3320562

[59] Netzer M, Kugler KG, Müller LA, Weinberger KM, Graber A, Baumgartner C, et al A network-based feature selection approach to identify metabolic signatures in disease. J Theor Biol. 2012;310:216–22. 10.1016/j.jtbi.2012.06.003 22771628

[60] Teusink B, Passarge J, Reijenga CA, Esgalhado E, van der Weijden CC, Schepper M, et al Can yeast glycolysis be understood in terms of in vitro kinetics of the constituent enzymes? Testing biochemistry. European Journal of Biochemistry. 2000;267(17):5313–5329. 10951190 10.1046/j.1432-1327.2000.01527.x

[61] Andrianopoulos V, Wagers SS, Groenen MT, Vanfleteren LE, Franssen FM, Smeenk FW, Vogiatzis I, Wouters EF, Spruit MA; CIRO+ Rehabilitation Network. Characteristics and determinants of endurance cycle ergometry and six-minute walk distance in patients with COPD. BMC Pulm Med. 2014 5 31;14:97 10.1186/1471-2466-14-97 24885117 PMC4229855

[62] Holz O, Roepcke S, Lauer G, Elmlinger M, Lahu G, Hohlfeld JM. Exercise Challenge Amplifies Differences In Metabolomic Signals Between Healthy Smokers And Smokers With COPD (gold2). Am J Respir Crit Care Med. 2014;189:A5952.

[63] Seggev JS, Thornton WH Jr, Edes TE. Serum leukotriene B4 levels in patients with obstructive pulmonary disease. Chest. 1991 2;99(2):289–91. 1846571 10.1378/chest.99.2.289

[64] Verhoeven GT, Garrelds IM, Hoogsteden HC, Zijlstra FJ. Effects of fluticasone propionate inhalation on levels of arachidonic acid metabolites in patients with chronic obstructive pulmonary disease. Mediators Inflamm. 2001 2;10(1):21–6. 11324900 10.1080/09629350123056 PMC1781690

[65] Dagouassat M, Gagliolo JM, Chrusciel S, Bourin MC, Duprez C, Caramelle P, Boyer L, Hue S, Stern JB, Validire P, Longrois D, Norel X, Dubois-Randé JL, Le Gouvello S, Adnot S, Boczkowski J. The cyclooxygenase-2-prostaglandin E2 pathway maintains senescence of chronic obstructive pulmonary disease fibroblasts. Am J Respir Crit Care Med. 2013 4 1;187(7):703–14. 10.1164/rccm.201208-1361OC 23328527

[66] An J, Li JQ, Wang T, Li XO, Guo LL, Wan C, Liao ZL, Dong JJ, Xu D, Wen FQ. Blocking of thromboxane A2 receptor attenuates airway mucus hyperproduction induced by cigarette smoke. Eur J Pharmacol. 2013 3 5;703(1–3):11–7. 10.1016/j.ejphar.2013.01.042 23399768

[67] Pina IL, Balady GJ, Hanson P, Labovitz AJ, Madonna DW, Myers J. Guidelines for Clinical Exercise Testing Laboratories A Statement for Healthcare Professionals From the Committee on Exercise and Cardiac Rehabilitation, American Heart Association. Circulation. 1995;91(3):912–921. 7828326 10.1161/01.cir.91.3.912

[68] Myers J, Arena R, Franklin B, Pina I, Kraus WE, McInnis K, et al Recommendations for Clinical Exercise Laboratories A Scientific Statement From the American Heart Association. Circulation. 2009;119(24):3144–3161. 10.1161/CIRCULATIONAHA.109.192520 19487589

[69] Driss T, Vandewalle H. The measurement of maximal (anaerobic) power output on a cycle ergometer: a critical review. BioMed research international. 2013;2013:589361 10.1155/2013/589361 24073413 PMC3773392

[70] Weinberger KM. Einsatz von Metabolomics zur Diagnose von Stoffwechselkrankheiten. (Translation: Metabolomics in diagnosing metabolic diseases.) Ther Umsch. 2008;65(9):487–91. 10.1024/0040-5930.65.9.487 18791962

[71] Astarita G, Langridge J. An emerging role for metabolomics in nutrition science. J Nutrigenet Nutrigenomics. 2013;6(4–5):181–200. 10.1159/000354403 24009004

[72] Wishart DS. Quantitative metabolomics using NMR. TrAC Trends in Analytical Chemistry. 2008;27(3):228–237.

[73] Dettmer K, Aronov PA, Hammock BD. Mass spectrometry-based metabolomics. Mass spectrometry reviews. 2007;26(1):51–78. 16921475 10.1002/mas.20108 PMC1904337

[74] Ramsay SL, Guggenbichler W, Weinberger KM, Graber A, Stoeggl WM (Inventors). Biocrates Life Sciences AG (Assignee). Device for quantitative analysis of a drug or metabolite profile. US patent 20070003965. Published 2007 Jan 4.

[75] Ramsay SL, Stoeggl WM, Weinberger KM, Graber A, Guggenbichler W (Inventors). Biocrates Life Sciences AG (Assignee). Apparatus and method for analyzing a metabolite profile. US patent 20070004044. Published 2007 Jan 4.

[76] Roberts LD, Souza AL, Gerszten RE, Clish CB. Targeted metabolomics. Current Protocols in Molecular Biology. 2012;30–2. 22470063 10.1002/0471142727.mb3002s98 PMC3334318

[77] Claeys M, Muscettola G, Markey SP. Simultaneous measurement of imipramine and desipramine by selected ion recording with deuterated internal standards. Biomed Mass Spectrom. 1976;3(3):110–6. 990418 10.1002/bms.1200030304

[78] Cui X, Churchill GA. Statistical tests for differential expression in cDNA microarray experiments. Genome Biol. 2003;4(4):210 12702200 10.1186/gb-2003-4-4-210 PMC154570

[79] Li W. Volcano plots in analyzing differential expressions with mRNA microarrays. Journal of bioinformatics and computational biology. 2012;10(06). 10.1142/S0219720012310038 23075208

[80] Xia J, Psychogios N, Young N, Wishart DS. MetaboAnalyst: a web server for metabolomic data analysis and interpretation. Nucleic acids research. 2009;37(suppl 2):W652–W660. 19429898 10.1093/nar/gkp356 PMC2703878

[81] Sana TR, Fischer S, Wohlgemuth G, Katrekar A, Jung KH, Ronald PC, et al Metabolomic and transcriptomic analysis of the rice response to the bacterial blight pathogen Xanthomonas oryzae pv. oryzae. Metabolomics. 2010;6(3):451–465. 20676379 10.1007/s11306-010-0218-7 PMC2899020

[82] Patti GJ, Tautenhahn R, Rinehart D, Cho K, Shriver LP, Manchester M, et al A view from above: cloud plots to visualize global metabolomic data. Analytical chemistry. 2013;85(2):798–804. 10.1021/ac3029745 23206250 PMC3716252

[83] Pearson K. Contributions to the mathematical theory of evolution. II. Skew variation in homogeneous material. Philosophical Transactions of the Royal Society of London A: Mathematical, Physical and Engineering Sciences. 1895;186:343–414.

[84] Wilk MB, Gnanadesikan R. Probability plotting methods for the analysis of data. Biometrika. 1968;55(1):1–17. 5661047

[85] Shapiro SS, Wilk MB. An analysis of variance test for normality (complete samples). Biometrika. 1965;52(3–4):591–611.

[86] Razali NM, Wah YB. Power comparisons of shapiro-wilk, kolmogorov-smirnov, lilliefors and anderson-darling tests. Journal of Statistical Modeling and Analytics. 2011;2(1):21–33.

[87] Ghasemi A, Zahediasl S. Normality tests for statistical analysis: a guide for non-statisticians. Int J Endocrinol Metab. 2012;10(2):486–9. 10.5812/ijem.3505 23843808 PMC3693611

[88] Wilcoxon F. Individual comparisons by ranking methods. Biometrics Bulletin. 1945;1(6):80–83.

[89] Benjamini Y, Hochberg Y. Controlling the false discovery rate: a practical and powerful approach to multiple testing. Journal of the Royal Statistical Society Series B. 1995;57:289–300.

[90] Yanagimoto T, Yamamoto E. Estimation of safe doses: critical review of the hockey stick regression method. Environmental health perspectives. 1979;32:193 540593 10.1289/ehp.7932193 PMC1637920

[91] Barrowman NJ, Myers RA. Still more spawner-recruitment curves: the hockey stick and its generalizations. Canadian Journal of Fisheries and Aquatic Sciences. 2000;57(4):665–676.

[92] Gan F, Ruan G, Mo J. Baseline correction by improved iterative polynomial fitting with automatic threshold. Chemometrics and Intelligent Laboratory Systems. 2006;82(1):59–65.

[93] Bao Q, Feng J, Chen F, Mao W, Liu Z, Liu K, et al A new automatic baseline correction method based on iterative method. Journal of Magnetic Resonance. 2012;218:35–43. 10.1016/j.jmr.2012.03.010 22578553

[94] Joosen RV, Kodde J, Willems LA, Ligterink W, van der Plas LH, Hilhorst HW. germinator: a software package for high-throughput scoring and curve fitting of Arabidopsis seed germination. The Plant Journal. 2010;62(1):148–159. 10.1111/j.1365-313X.2009.04116.x 20042024

[95] Lommen A, Gerssen A, Oosterink JE, Kools HJ, Ruiz-Aracama A, Peters RJ, et al Ultra-fast searching assists in evaluating sub-ppm mass accuracy enhancement in U-HPLC/Orbitrap MS data. Metabolomics. 2011;7(1):15–24. 21461040 10.1007/s11306-010-0230-y PMC3040356

[96] Wei X, Sun W, Shi X, Koo I, Wang B, Zhang J, et al MetSign: A computational platform for high-resolution mass spectrometry-based metabolomics. Analytical chemistry. 2011;83(20):7668–7675. 10.1021/ac2017025 21932828 PMC3196362

[97] Witten IH, Frank E, Hall MA. Data mining—practical machine learning tools and techniques 3rd ed. Burlington, MA: Elsevier; 2011.

