# Supplementary figures and images for: Modeling and Classification of Kinetic Patterns of Dynamic Metabolic Biomarkers in Physical Activity
Source: PLoS Comput Biol. 2015 Aug 28;11(8):e1004454. doi: 10.1371/journal.pcbi.1004454 (PMC4552566; doi:10.1371/journal.pcbi.1004454)

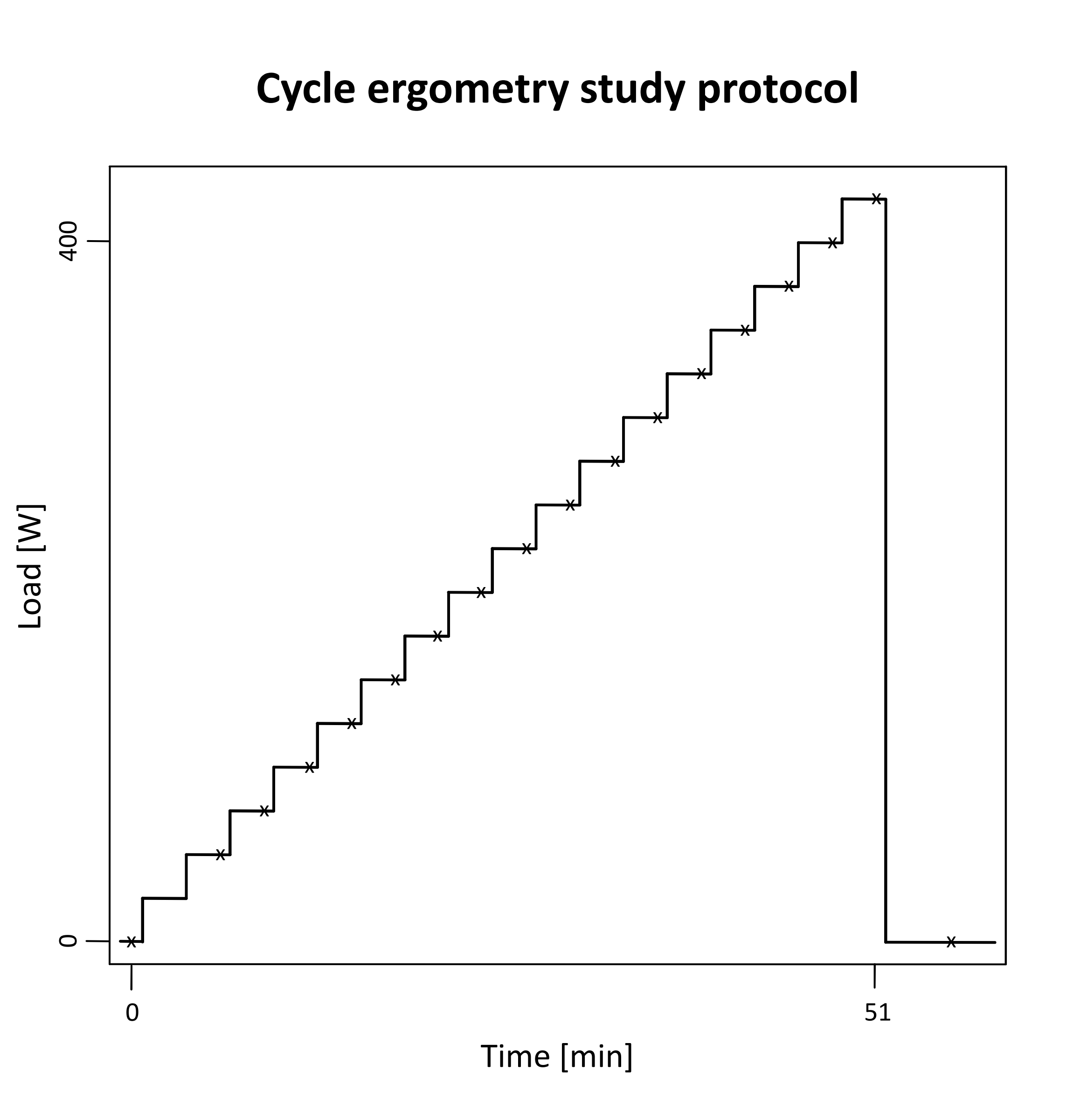

Supplement: S1 Fig — Study protocol of the cycle ergometry performance test, exemplarily shown for the maximum workload level of 425 W. Workload is increased by 25 W every 3 minutes (25 W level is skipped). Blood samples are taken at rest, with every new workload level, and after a recovery period of five minutes after the individual maximum workload. (TIF) [file pcbi.1004454.s001.tif]

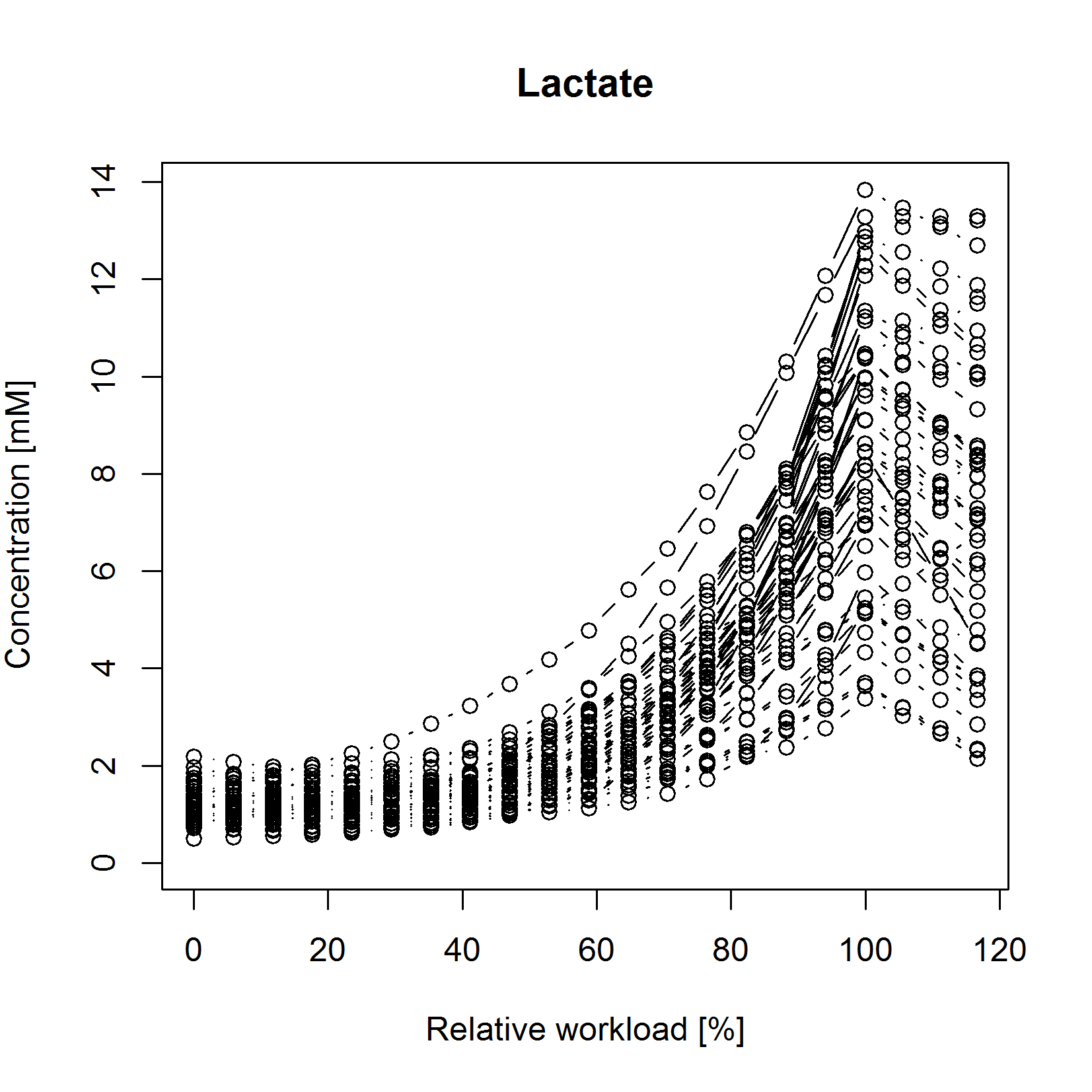

Supplement: S2 Fig — Lactate concentration curves of the 47 analyzed individuals. To ensure comparability of individual workload curves, concentration curves are rescaled and values interpolated (see section Data preprocessing). (TIF) [file pcbi.1004454.s002.tif]

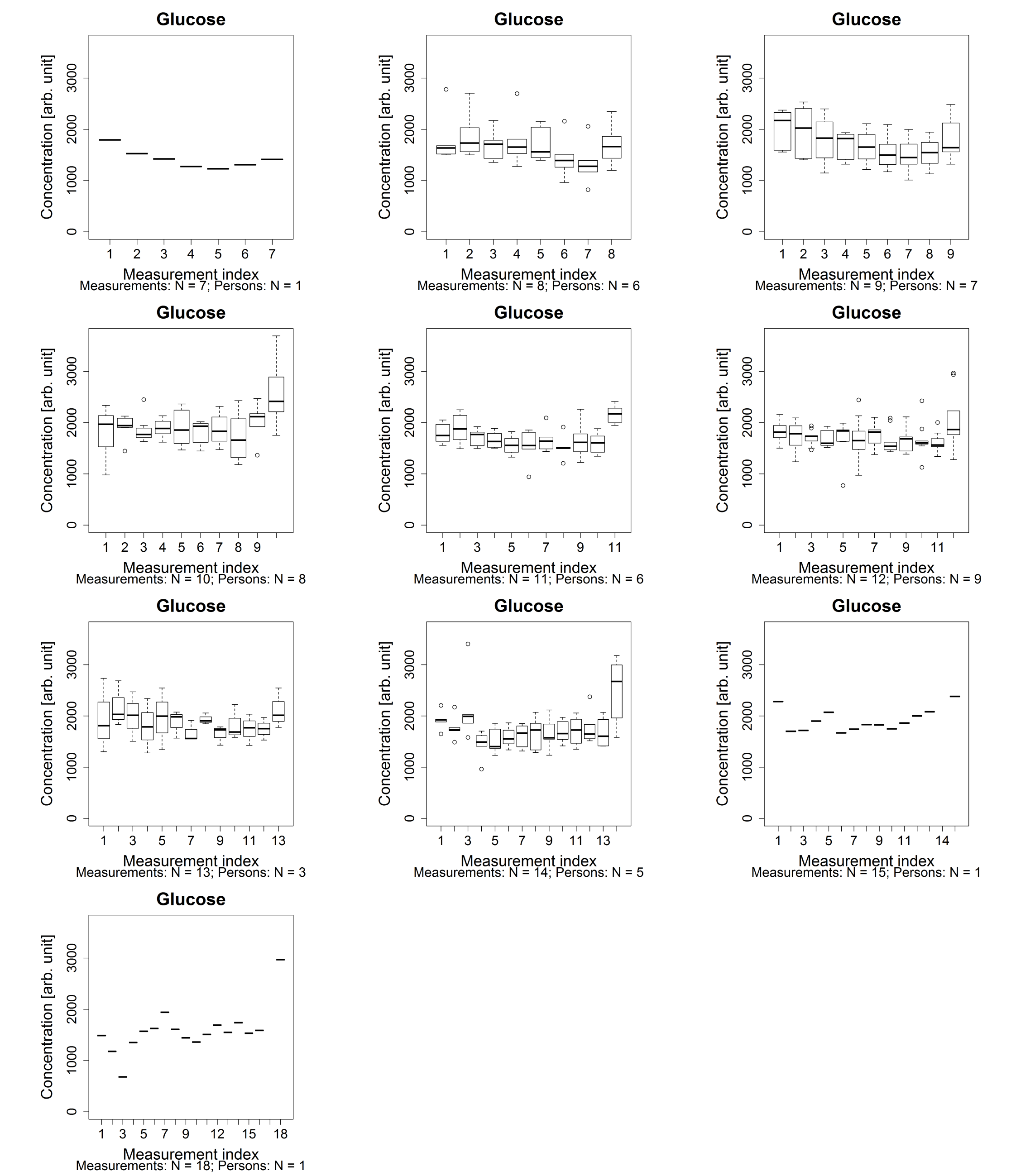

Supplement: S3 Fig — Box plots for descriptive analysis of raw data. Data are visualized by grouping persons into subgroups reaching the same maximal workload level. The minimum number of measurements is seven (at 150 W of individual’s maximum load) and the maximum number of measurements is 18 (at 425 W). (TIF) [file pcbi.1004454.s003.tif]

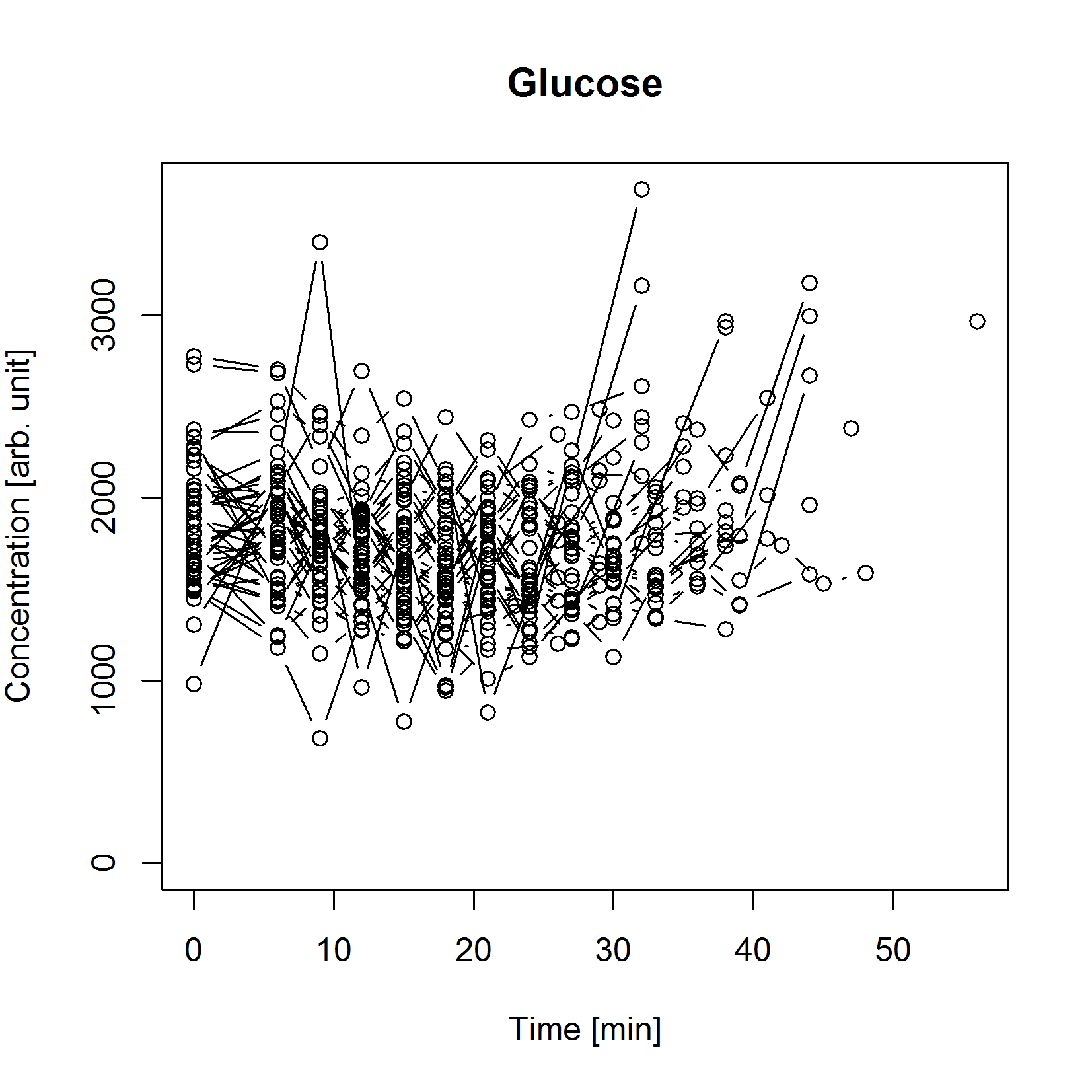

Supplement: S4 Fig — Visualization of raw concentration curves for glucose, where data are visualized with regard to time points of measurement. The shortest individual workload curve ends after approx. 23 minutes, the longest individual workload curve after approx. 56 minutes. (TIF) [file pcbi.1004454.s004.tif]

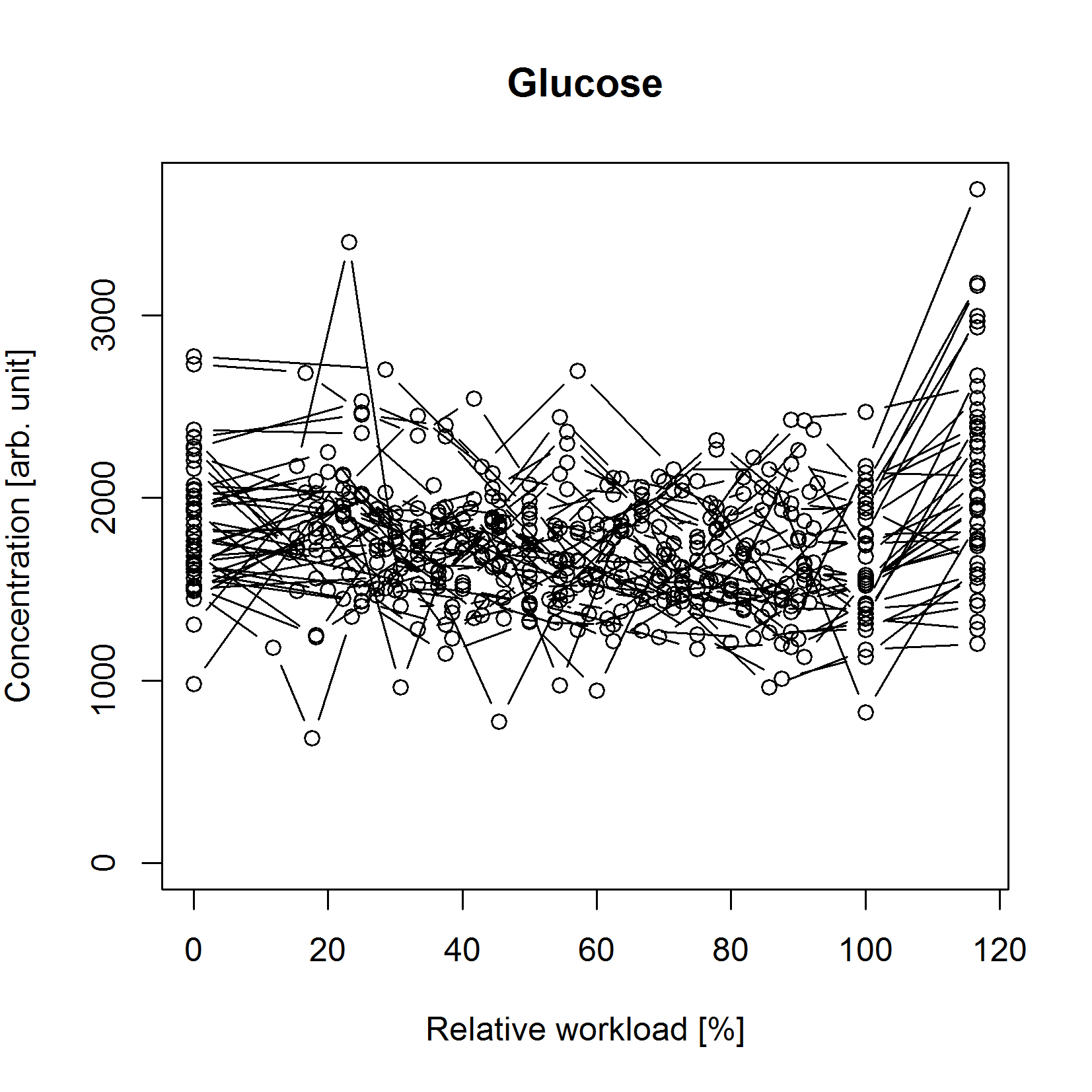

Supplement: S5 Fig — To ensure comparability of differing individual workload curves, a rescaling of data is performed referring to the individual maximum physical load as 100%. The values for recovery are rescaled with respect to the median recovery value of 117%. (TIF) [file pcbi.1004454.s005.tif]

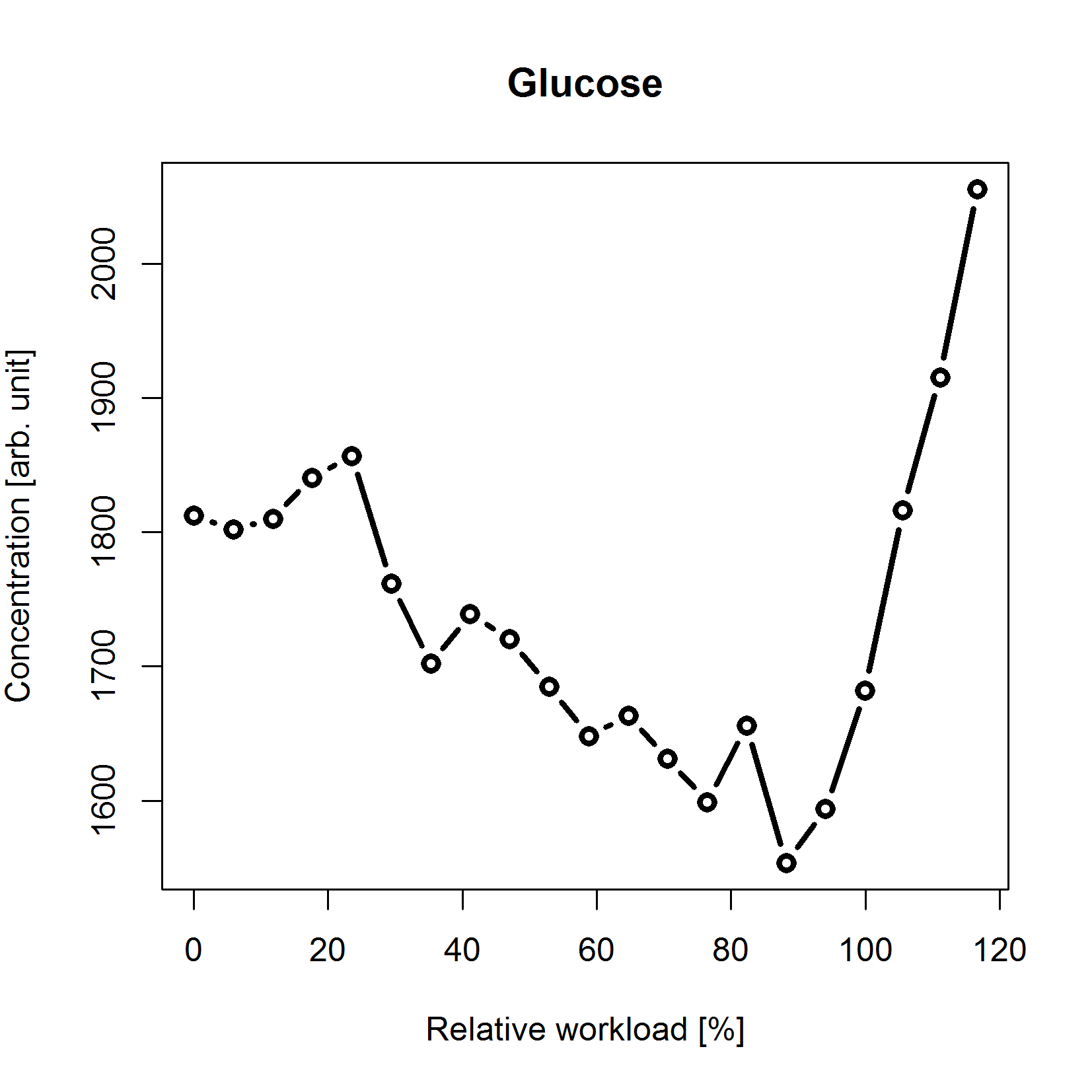

Supplement: S6 Fig — Glucose median concentration values extracted from interpolated concentration curves, which are used as a basis for mathematical modeling. (TIF) [file pcbi.1004454.s006.tif]

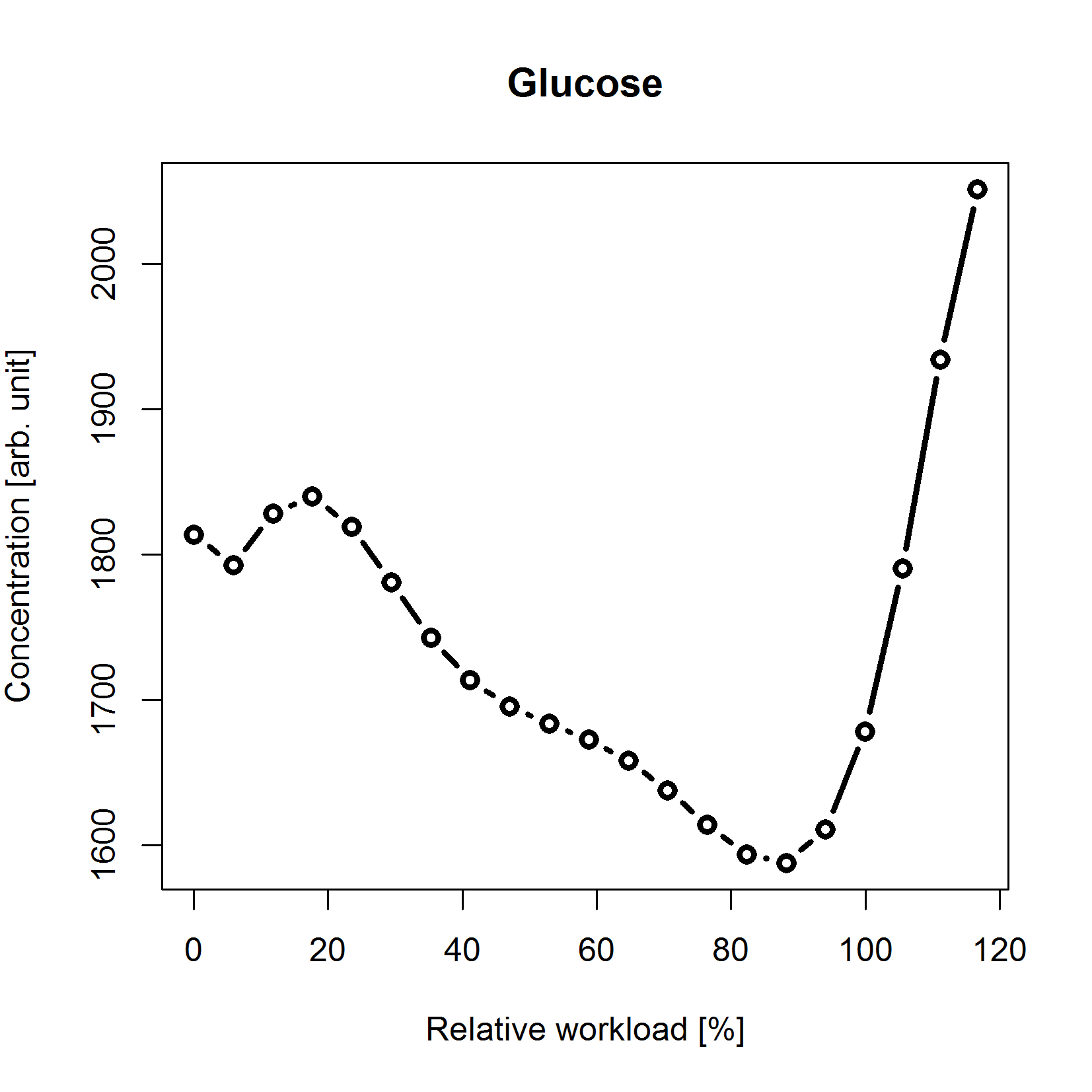

Supplement: S7 Fig — Mathematical modeling by fitting a polynomial of 9th degree to median concentration values, extracted from interpolated concentration values. (TIF) [file pcbi.1004454.s007.tif]

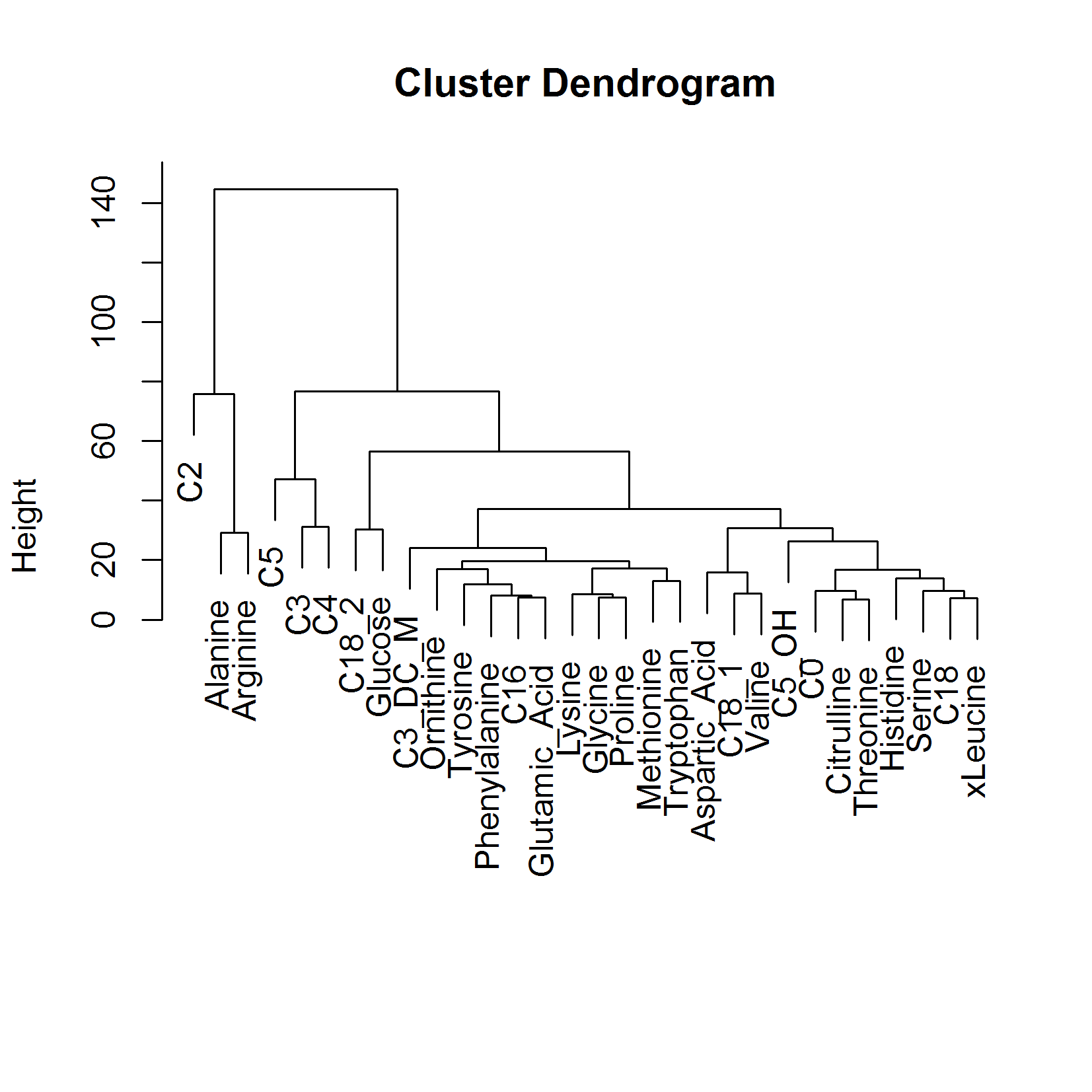

Supplement: S8 Fig — Dendrogram of hierarchical clusters analysis. Metabolites acetylcarnitine (C2), valerylcarnitine (C5), propionylcarnitine (C3), butyrylcarnitine (C4), octadecadienylcarnitine (C18:2), glucose, alanine, and arginine are separated at a high cut-off level. (TIF) [file pcbi.1004454.s008.tif]

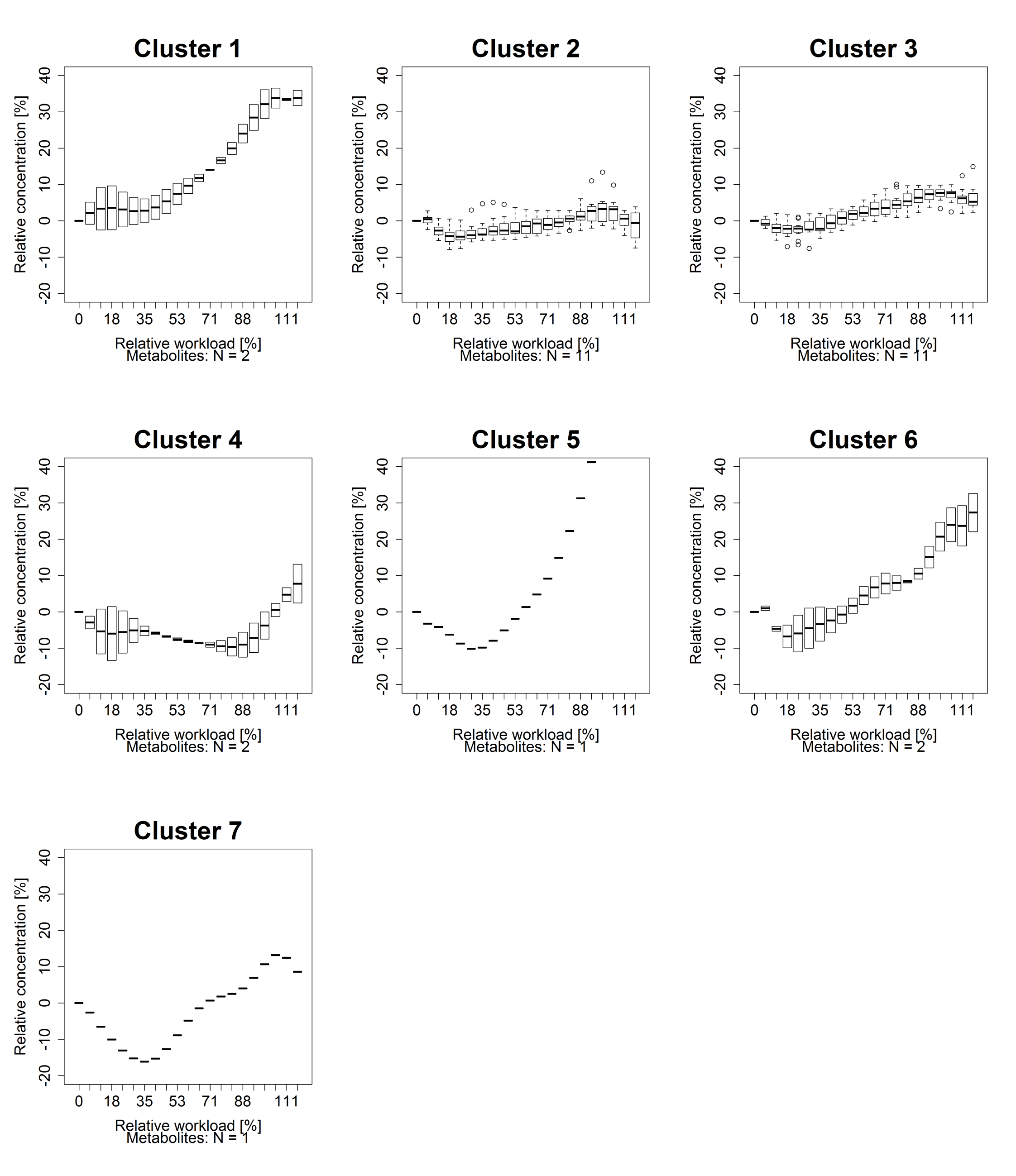

Supplement: S9 Fig — For each cluster a box plot for a descriptive representation is generated. Median values are selected from the concentration curves of each cluster, serving as basis for the specification of kinetic shape templates. (TIF) [file pcbi.1004454.s009.tif]

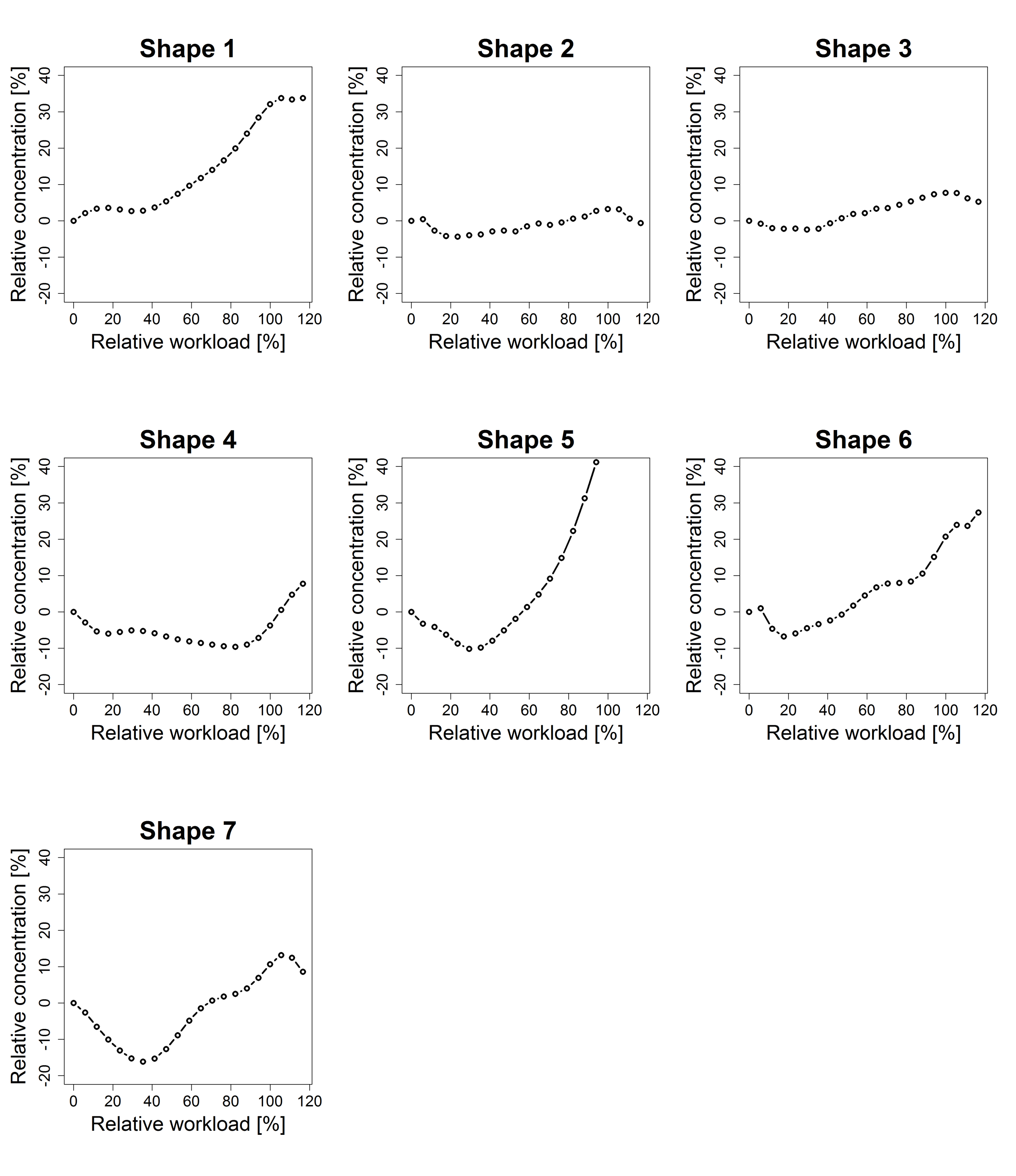

Supplement: S10 Fig — Median concentration curves of all metabolite clusters (identified through hierarchical cluster analysis), serving as basis for the specification of kinetic shape templates. (TIF) [file pcbi.1004454.s010.tif]
